# Sequence learning modulates neural responses and oscillatory coupling in human and monkey auditory cortex

**DOI:** 10.1371/journal.pbio.2000219

**Published:** 2017-04-25

**Authors:** Yukiko Kikuchi, Adam Attaheri, Benjamin Wilson, Ariane E. Rhone, Kirill V. Nourski, Phillip E. Gander, Christopher K. Kovach, Hiroto Kawasaki, Timothy D. Griffiths, Matthew A. Howard, Christopher I. Petkov

**Affiliations:** 1 Institute of Neuroscience, Newcastle University, Newcastle upon Tyne, United Kingdom; 2 Centre for Behaviour and Evolution, Newcastle University, Newcastle upon Tyne, United Kingdom; 3 Human Brain Research Laboratory, Department of Neurosurgery, The University of Iowa, Iowa City, Iowa, United States of America; 4 Wellcome Trust Centre for Neuroimaging, University College London, London, United Kingdom; Max-Planck-Institut für Kognitions- und Neurowissenschaften, Germany

## Abstract

Learning complex ordering relationships between sensory events in a sequence is fundamental for animal perception and human communication. While it is known that rhythmic sensory events can entrain brain oscillations at different frequencies, how learning and prior experience with sequencing relationships affect neocortical oscillations and neuronal responses is poorly understood. We used an implicit sequence learning paradigm (an “artificial grammar”) in which humans and monkeys were exposed to sequences of nonsense words with regularities in the ordering relationships between the words. We then recorded neural responses directly from the auditory cortex in both species in response to novel legal sequences or ones violating specific ordering relationships. Neural oscillations in both monkeys and humans in response to the nonsense word sequences show strikingly similar hierarchically nested low-frequency phase and high-gamma amplitude coupling, establishing this form of oscillatory coupling—previously associated with speech processing in the human auditory cortex—as an evolutionarily conserved biological process. Moreover, learned ordering relationships modulate the observed form of neural oscillatory coupling in both species, with temporally distinct neural oscillatory effects that appear to coordinate neuronal responses in the monkeys. This study identifies the conserved auditory cortical neural signatures involved in monitoring learned sequencing operations, evident as modulations of transient coupling and neuronal responses to temporally structured sensory input.

## Author summary

While natural environments constantly change, certain events can predict the future occurrence of others. Learning ordering relationships is vital for animal perception and human communication, yet how such learning and prior experience affect the brain remains poorly understood. We set out to understand how learning relationships between words modifies neuronal responses in both humans and monkeys. Using an implicit learning paradigm, we exposed human subjects and monkeys to sequences of nonsense speech sounds that followed certain rule-based ordering relationships (an “artificial grammar”). We then recorded neural responses directly from the auditory cortex in both species in response to sequences that were either consistent with the artificial grammar or created illegal ordering transitions between elements in a sequence. We found that learned ordering relationships modulate a diversity of neural responses (some of which coordinate in similar ways) at the scale of populations of neurons in both species. Our experiments in monkeys also revealed that this scale of neural processing is related to single neurons, the fundamental processing unit in the brain. This study reveals the conserved neuronal signatures of the auditory cortex involved in monitoring learned sequencing operations, which mechanistically inform and extend ideas on how the brain predicts the sensory world.

## Introduction

Natural environments are dynamic and constantly changing, yet certain sensory events can predict the occurrence of others. For any animal to adapt and survive in its environment requires that its brain establish and monitor the predictability of ordering relationships between sensory events, a process that is impaired in neurodevelopmental and other disorders [1–3]. It is known that neural oscillations at certain frequencies can entrain to rhythmic sensory inputs, regulating the excitability of neuronal populations [4–13]. However, how learning and prior experience with ordering relationships affect neural oscillations and neuronal responses in the sensory neocortex is poorly understood.

Sequence learning paradigms can be used to comparatively test the sensitivity of human and nonhuman animals to temporal order in sequences of sensory items [14–21]. Typically, such experiments begin with an exposure period, during which the participant experiences the regularities between the sensory items in a sequence—for example, listening to a string of legal (“consistent”) sequences of sounds generated by a rule-based system (i.e., an “artificial grammar”). The exposure phase is thought to elicit implicit learning of the ordering relationships between the sensory items, a form of relational knowledge that does not require perceptual awareness about what was learned [22]. The exposure period is followed by a testing period in which the participant is presented with novel, consistent sequences and “violation” sequences that have illegal transitions not experienced during exposure. Differential responses to different types of sequence ordering relationships can provide insights into the participant’s sensitivity and learning strategy. There is growing evidence that following exposure to representative legal sequences, humans and various species of nonhuman animals can recognize ordering relationships between events in a sequence, and there is considerable interest in understanding whether temporal sequence processing capacities are an evolutionary precursor substrate upon which human language evolved [14–19, 21].

Although theoretical models and general comparisons across species point to broadly evolutionarily conserved neural oscillatory processes [6, 7, 23–25], there is a paucity of direct comparative evidence in humans and animal models. Direct intracranial recordings can occasionally be obtained in humans being monitored for surgery, when the coverage for clinical monitoring overlaps with the research question. However, to date there has been little common ground in terms of recording sites or tasks across the species, making it difficult to extrapolate insights on neural mechanisms from animal models to humans. Moreover, certain neural processes involved in segmenting human speech and language [7–9, 26–29] are unlikely to have direct correspondences in nonhuman animals, given that human language is unique in the animal kingdom. To test for and establish whether neural processes are evolutionarily conserved requires direct comparative neurobiological data obtained under similar tasks.

The goal of this study was to identify and compare the neural oscillatory signatures in the monkey and human auditory cortex in response to sequences of nonsense words and to test whether and how neural responses and oscillations, including single-neuron responses in the monkeys, are modified by the learned between-word relationships established during the preceding exposure period. Based on neurobiological models of auditory cortical oscillatory processing of speech sounds [26] or sensory-driven entrainment of low-frequency neural oscillations [5, 6], we hypothesized that nonsense word sequences consisting of spectrotemporally complex sounds would elicit low-frequency phase and gamma amplitude coupling in the human and monkey auditory cortex. Whether the form of neural oscillations or coupling would be similar or different across the species was unclear. It was also difficult to predict how the learned sequencing relationships would affect cortical oscillations or coupling, although some form of impact on low frequency oscillations might be expected [6, 25, 27, 28]. Moreover, we expected that phase–amplitude coupling (PAC), which can capture extrinsic influences [9, 24–28], would show evidence of either occurring in tandem or leading corresponding effects on local neurons (i.e., single neurons or populations of neurons, as measured by local field potential power at higher frequencies) during sequence processing. The results show comparable low-frequency (delta and/or theta) phase and high-gamma amplitude coupling in response to the nonsense words in population neural activity recorded directly from the human and monkey auditory cortex, which was robust even with the stimulus-driven response removed from analysis. The strength of this form of nested coupling was strongly modulated by the temporal sequencing regularities in the sequences, as established by the prior exposure period to the ordering relationships. Sequence-processing effects on oscillatory coupling and single-neuron spiking activity occurred in tandem but preceded effects on local field potential power. Sensitivity to the sequence ordering relationships was also time sensitive, with, for instance, modulations of oscillatory coupling in response to sequencing relationships consistent with the artificial grammar occurring prior to further modulations of oscillatory coupling in response to violations of sequence ordering relationships. The results inform predictive coding models. This study demonstrates that learned sequence ordering relationships modulate neural oscillations in ways that are found to be remarkably similar in the human and monkey auditory cortex and that such oscillatory coupling appears to coordinate local neuronal responses in the primate auditory cortex.

## Results

We used an “artificial grammar” learning paradigm that generates sequences of nonsense words with considerable variability in the transitions between elements (S1 Fig). This paradigm has been used to identify differences in the sequence processing capabilities of different nonhuman primate species [19] but, critically, appears to be processed comparably by humans and macaques [20]. We used nonsense words because they contain all the spectrotemporal components of speech needed to elicit theta–gamma coupling in the human auditory cortex [25, 26] but minimize human lexical–semantic recognition. This made for a closer cross-species comparison since the monkeys are likely to treat the nonsense words as complex sounds and/or vocalizations, but they would not be able to attach meaning to them. Since the transitions between the nonsense word stimuli occur at a lower frequency (~1.8 Hz) than the within-word acoustic features, this allowed us to evaluate whether and how the learned contingencies between the nonsense words would affect the form of neural oscillations seen in both monkeys and humans.

Eye-tracking measurements of orienting behavior with the two monkeys that participated in these experiments have previously been used to confirm that the macaques produce longer looking responses to sequences that violated the between-word ordering relationships than to novel, “consistent” sequences that followed the legal ordering relationships [19]. Moreover, macaques and humans show comparable patterns of behavioral responses to the regularities in the sequences, in particular for adjacent relationships between the nonsense words in the sequences [21]. During a follow-up visit after the surgical monitoring period, one of the human subjects in this study participated in our behavioral testing paradigm. The results confirmed the subject’s sensitivity to the sequencing violations (S2 Fig).

**Fig 1:**
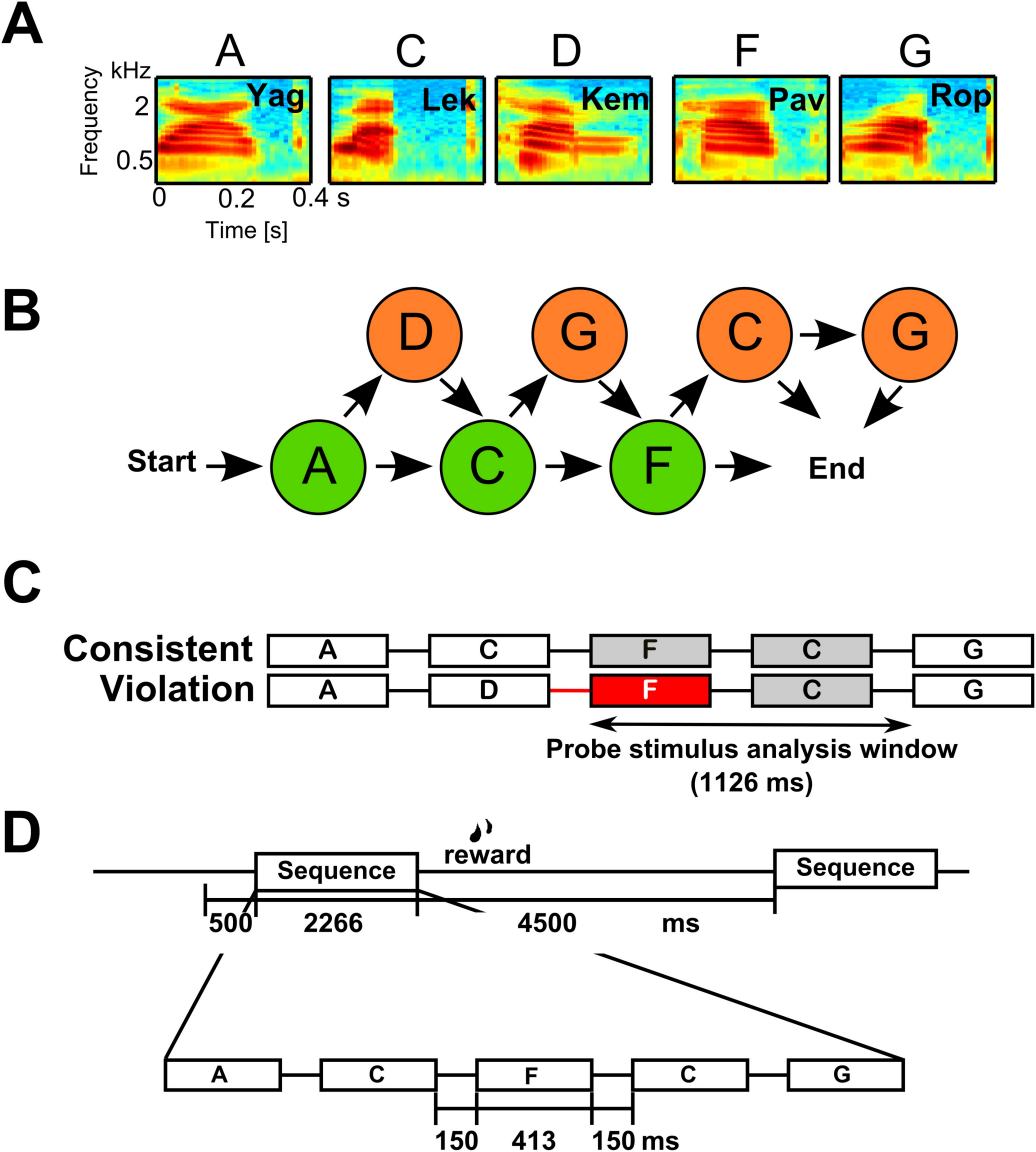
Associative sequence learning paradigm. A. Spectrograms of the five nonsense word elements used in this study. B. The paradigm used is based on Wilson et al. [19] using the artificial grammar of Saffran et al. [30]. It consists of required (green) and optional (orange) nonsense word elements. In the illustration, following any of the arrows from start to end generates a legal “consistent” sequence. C. Example consistent and matching violation sequence comparison pair. The red box highlights the first illegal sound element in the sequence. This illustrates how, in the monkeys, local field potential (LFP) and single-unit activity (SUA) data were analyzed using a 1,126 ms–long analysis window (“probe stimulus analysis window” denoted by the horizontal arrow) to include responses to the same two acoustically identical nonsense words (here the F and C elements, gray and red) in the two sequencing conditions. See S1 Fig for all comparison sequence pairs used. D. Illustrated monkey testing trial time course. Sequences consisted of nonsense words (each 413 ms–long) separated by a 150-ms interstimulus interval (ISI). A sequence was initiated by the monkey fixating on a visual spot on the monitor screen for at least 500 ms, and each sequence was separated by at least a 4,500-ms intertrial interval (ITI). See Materials and methods for details on the human experiment using the same materials.

We first presented the monkeys with the exposure set of representative consistent sequences for 30 min (Fig 1 and S1 Fig). This was followed by the testing period, during which we recorded single-unit activity (SUA) from a total of 187 neurons (M1: *n* = 126; M2: *n* = 61) and 145 local field potentials (LFPs) (M1: *n* = 90; M2: *n* = 55). The recordings were made from tonotopically localized regions of the auditory cortex (involving primary and surrounding auditory belt regions) as the animals listened to the testing sequences (S1 Fig). The testing sequences were either consistent with the ordering relationships previously heard during exposure or violated a specific ordering relationship. Critically, the neurobiological effects reported for the sequencing relationships cannot be attributed to acoustic features, since identical nonsense word elements were directly compared in matched pairs of consistent and violation sequences. In other words, the analyses focused on the same acoustical elements (probe stimulus period), prior to which there was either a violation or no violation, which we refer to as the “sequencing context” (Fig 1C, S1 Fig).

**Fig 2:**
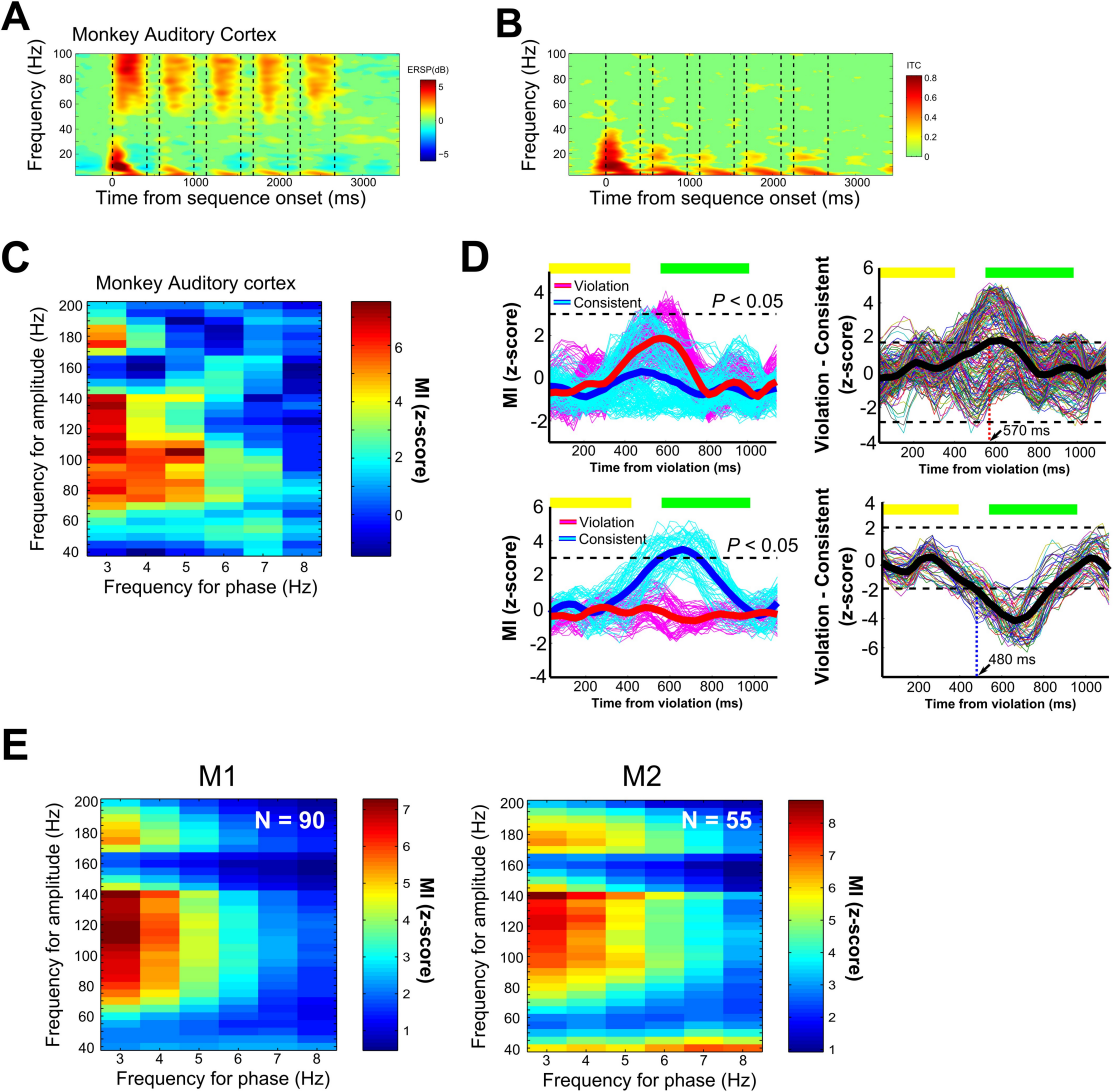
Monkey phase–amplitude coupling in response to the nonsense words and between-word transitions. A. Time-frequency representation of local field potential (LFP) cortical responses in the monkey auditory cortex to the nonsense words (all eight sequences). Shown is the event-related spectral perturbation (ERSP). The color scale indicates power (in dB) at a given frequency and time relative to the 500-ms baseline prior to sequence onset. B. Phase consistency was calculated using intertrial phase coherence (ITC). Bootstrap statistics (*p* < 0.01) were computed using the same baseline used to calculate the ERSP, and nonsignificant points were colored in green for both ERSP and ITC. C. Exemplary phase–amplitude coupling (PAC) in response to the nonsense words for the same data shown in (A) and (B). The MI values were z-scaled and shown at each combination of frequency for phase in the *x*-axis and frequency of amplitude in the *y*-axis. D. The left panels show the modulation index (MI) time course of PAC during the analysis window, extracted from the combinations of amplitude and phase pairs in response to the nonsense words that were above the statistical threshold (*p* < 0.05, Bonferroni correction). The results shown here are for the same exemplary LFP site shown in A–C. The thick lines denote the average PAC response across all significant phase and amplitude pairs, and the thin lines show the coupling strength for all phase–amplitude pairs. The horizontal yellow and green bars above the response curve identify the time of occurrence of the elements in the two types of sequences after the violation onset in the violation sequence or the acoustically identical elements in the corresponding consistent sequence. The right two panels show difference plots of the time course of oscillatory coupling in response to the violation versus the corresponding consistent sequence. The thick line denotes the average difference calculated from the data on the left. The horizontal dotted lines denote the CI significance threshold. The vertical dotted line identifies the latency of the first significant effect. The top panels show an exemplary response with a violation-preferring sequencing context effect. The bottom panels show exemplary response with a consistent-preferring sequencing context effect. E. Average PAC in response to the nonsense words across all LFP sites in the monkeys (145 LFPs, *p* < 0.05, Bonferroni-corrected).

We first evaluated monkey auditory neural oscillatory responses to the nonsense words, irrespective of the sequencing context. Macaque neural responses showed evidence of low-frequency (including theta band) phase alignment and high-gamma power in response to the nonsense words. We observed significant event-related spectral perturbations (ERSPs) [31] in response to each of the nonsense words, which were prominent in theta (4–10 Hz), alpha (10–13 Hz), beta (13–30 Hz), and high-gamma (>50 Hz) frequency bands (Fig 2A). Significant increases in intertrial phase coherence (ITC) were also observed in theta, alpha, and beta frequency bands (bootstrap significance level, *p* < 0.01, Fig 2B). These phase (ITC) and power (ERSP) oscillatory responses to each of the sounds in the sequence substantiate that the core ingredients are in place to assess low-frequency phase and high-frequency amplitude coupling.

We next evaluated whether significant coupling between low-frequency phase and high-frequency amplitude occurs and, if so, what form such coupling takes and whether it can occur with the stimulus-induced response removed. We calculated PAC in the LFP signals using a modulation index (MI) to evaluate the strength of coupling between low-frequency phase (3–8 Hz) and high-frequency power (40–200 Hz). The vast majority of the LFP sites recorded from the monkey auditory cortex showed robust PAC responses to the nonsense words as low-frequency (3–8 Hz) phase coupling with gamma (>40 Hz) amplitude (141/145, 97%; M1: 88/90, 98%; M2: 53/55, 96%, *p* < 0.05, Bonferroni corrected; Fig 2C and 2E). Our approach ensured that the results cannot be easily attributed to stimulus-evoked responses since we removed the stimulus-driven component from contributing to the analyses (see Materials and methods, S3 Fig).

Next, to evaluate the impact of the sequencing context on theta–gamma coupling (i.e., oscillatory coupling in response to the nonsense words), the time course of MI values was extracted from the cluster of pixels showing PAC in response to the nonsense words under the two sequencing conditions (Fig 2D). For the 141 LFP sites that showed significant PAC effects in response to nonsense words, 53% (75 LFPs, M1: 47/88, 53%; M2: 28/53, 53%) showed that the sequencing context significantly modulated PAC coupling (*p* < 0.05, Bonferroni corrected; Fig 2D). The exemplary site in Fig 2D shows that violations of the sequencing order (i.e., a transition that never occurred during the exposure period) strongly modulated coupling between low-frequency phase and gamma amplitude at specific times from ~450 ms after the violation to ~700 ms; this is a time period covering the subsequent two sounds after the sequencing violation.

The modulation of PAC seemed to occur independently of any stimulus-evoked, ERP-driven, or time-locked ITC during the same probe stimulus period (see S3 Fig). This observation provides additional evidence that the observed modulation of PAC reflects a sequencing context–related effect rather than one that is purely stimulus driven. Out of 75 sites, the largest proportion (34/75; 45%) showed greater PAC responses to violation sequences. Interestingly, a considerable number of sites also showed greater PAC responses to consistent sequences (31/75; 41%) and responses to both types of sequencing contexts (10/75; 13%), the latter evident as significant deviations from chance variability in PAC for either context at different time points.

Interestingly, the form of low-frequency phase and high-gamma amplitude coupling observed in monkeys in this study resembles that obtained in previous intracranial results in humans [25]. However, it is difficult to directly compare our monkey results to this pioneering work in humans, as it relied on word recognition tasks that can only be conducted in humans. Furthermore, regions including and around the primary auditory cortex (Heschl’s gyrus [HG]) were not assessed, which would better correspond to the regions that were recorded here in the monkeys. For more direct cross-species comparisons, we recorded from two human patients being monitored for surgery who had depth electrodes in HG as part of their clinical treatment plan. We used auditory stimulation sequences identical to those in the monkey experiments for both exposure and testing (see Materials and methods).

**Fig 3:**
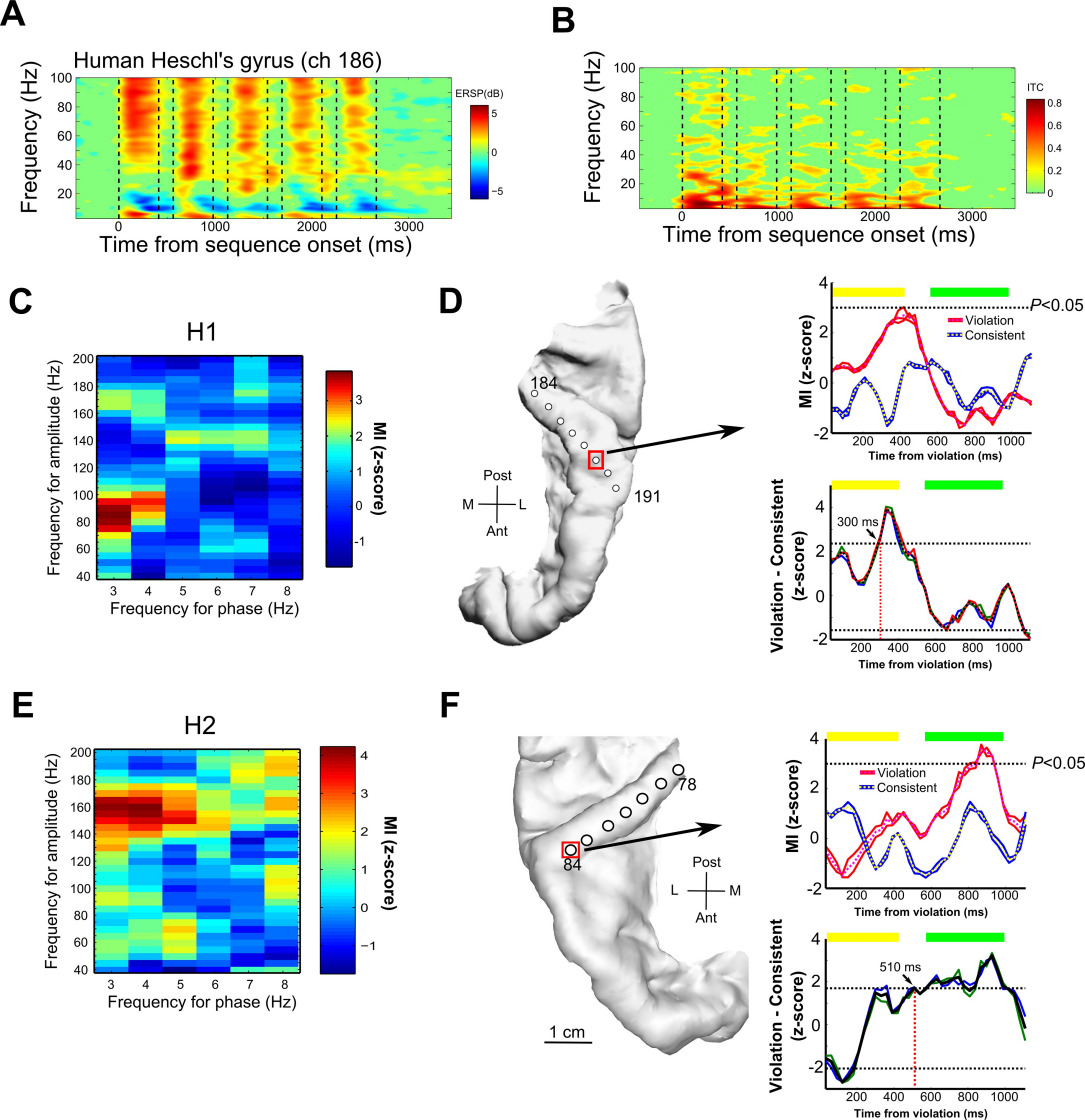
Human intracranial recordings from the auditory cortex in response to the nonsense words and sequencing relationships. Figure format is the same as in Fig 2 for the monkey results. A. Time-frequency representation of neuronal responses in an exemplary local field potential (LFP) recording site (channel 186) from Heschl’s gyrus (HG) in human subject 1 (H1). B. Intertrial phase coherence (ITC). C. Exemplary LFP phase–amplitude coupling (PAC) in response to nonsense words shown for site 186. D. Left: reconstructed image of the location of the depth electrode placement in the left HG for H1. Right: (top) time course of modulation index (MI) values above the significance threshold at the recording site identified by a red square in panel (D). (bottom) Difference plot of the time course of oscillatory coupling in response to the violation versus the corresponding consistent sequence. PAC showed a significant difference in response to the violation sequence at a latency of ~300ms (bootstrap statistics, *p* < 0.05). E. Exemplary LFP PAC in response to nonsense words shown for site 81 in H2. F. Left: depth electrode placement in the right HG in H2. Right: (top) time course of MI values above the significance threshold at the recording site, identified by the red square in panel (F). (Bottom) difference plot of the time course shown in the plot above with a sequencing-context sensitivity latency of 510 ms.

The human neural oscillations recorded in HG showed striking similarities to the results obtained from the monkey auditory cortex. We observed increased gamma power and ITC in the low-frequency range in response to the nonsense words (compare human Fig 3A and 3B to monkey Fig 2A and 2B). Moreover, 81% (13/16) of the contacts in human HG showed significant low-frequency phase and gamma amplitude coupling (*p* < 0.05, Bonferroni corrected) in response to the nonsense words (human subject 1 [H1]: 6/8 = 75%; H2: 7/8 = 88%; Fig 3C–3F). Out of 13 LFP recording sites in HG, 10 sites (77%) showed significant modulation of phase–amplitude coupling in response to the sequencing context (effects in at least one of the stimulus sequence pairs; H1: 5/6 = 75%; H2: 5/7 = 88%). This is seen as low-frequency-to-gamma coupling that is dynamically modulated over time after a sequencing violation (Fig 3D right panels, *p* < 0.05, Bonferroni-corrected; significant modulation as a function of the sequencing context). The presence of mixed types of context-dependent PAC responses (Fig 2D), as was seen in the monkey results, was also observed in the humans; out of 10 sites that showed significant context-dependent PAC responses, 30% (3/10) showed significant PAC in response to the violation sequences, 40% (4/10) showed greater response to the consistent sequence, and 30% (3/10) showed significant PAC modulation to both types of sequences. The observed PAC effects varied across the gamma frequency range and did not show clear topographical relationships in response to the two sequencing conditions across the recorded sites in the human or monkey auditory cortex (S1 Text, S4 Fig, S5 Fig and S6 Fig). Further, a control experiment in another human subject (H3) provides some support for the notion that effects on PAC are stronger when the participant experiences exposure to statistically structured sequences rather than those with unstructured transitions between the sound elements (S2 Text, S7 Fig, S1 Table and S2 Table).

### LFP power and SUA effects in relation to single-neuron responses

**Fig 4:**
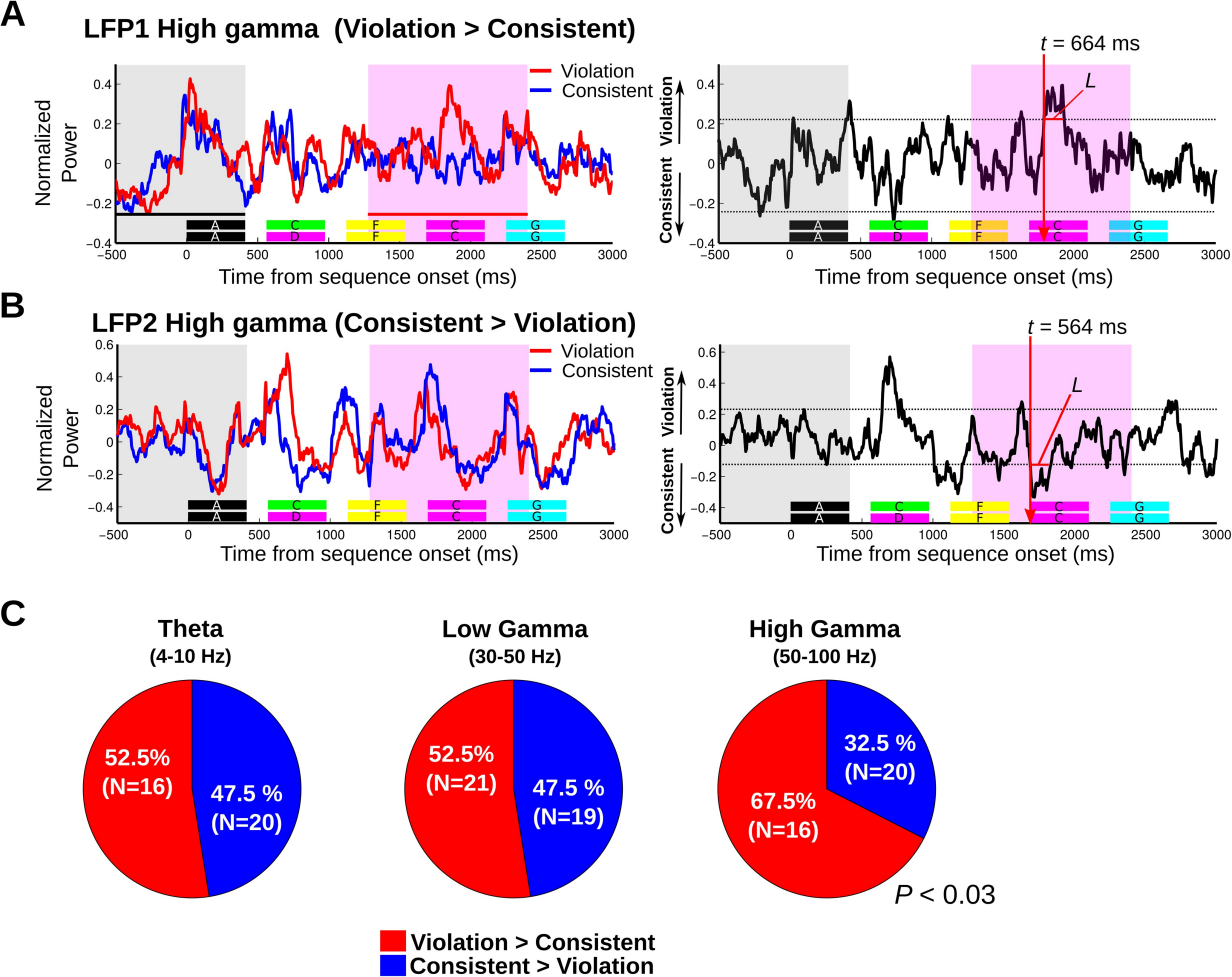
Exemplary monkey local field potential (LFP) response sensitivity to the sequencing context. A. (left) An exemplary LFP high-gamma response (50–100 Hz) to consistent (blue line) and violation sequences (red line), showing greater response to the violation sequence during the probe stimulus analysis window (pink window in left panel). The horizontal color keys below the response curves indicate each element of the consistent and the violation sequences. The gray area indicates the 913-ms baseline period (i.e., 500 ms prior to the sequence onset through to the end of the first “A” element). (Right) a difference response waveform was created by subtracting the grand average response to the consistent sequence from the grand average response to the violation sequence (“violation”–“consistent”). “*L*” is the length of the sequencing context–dependent response (i.e., response duration breach above the CIs) and “*t*” is the sensitivity latency, which is defined as the first time point that breached the CI. Significance of breach is based on permutation tests using both duration and magnitude criteria (see Materials and methods). B. An exemplary LFP high-gamma response (50–100 Hz) that is greater for the consistent sequence. C. Proportions of responses to sequences showing significant effects to either the consistent or violation conditions, subdivided by different LFP frequency bands.

**Fig 5:**
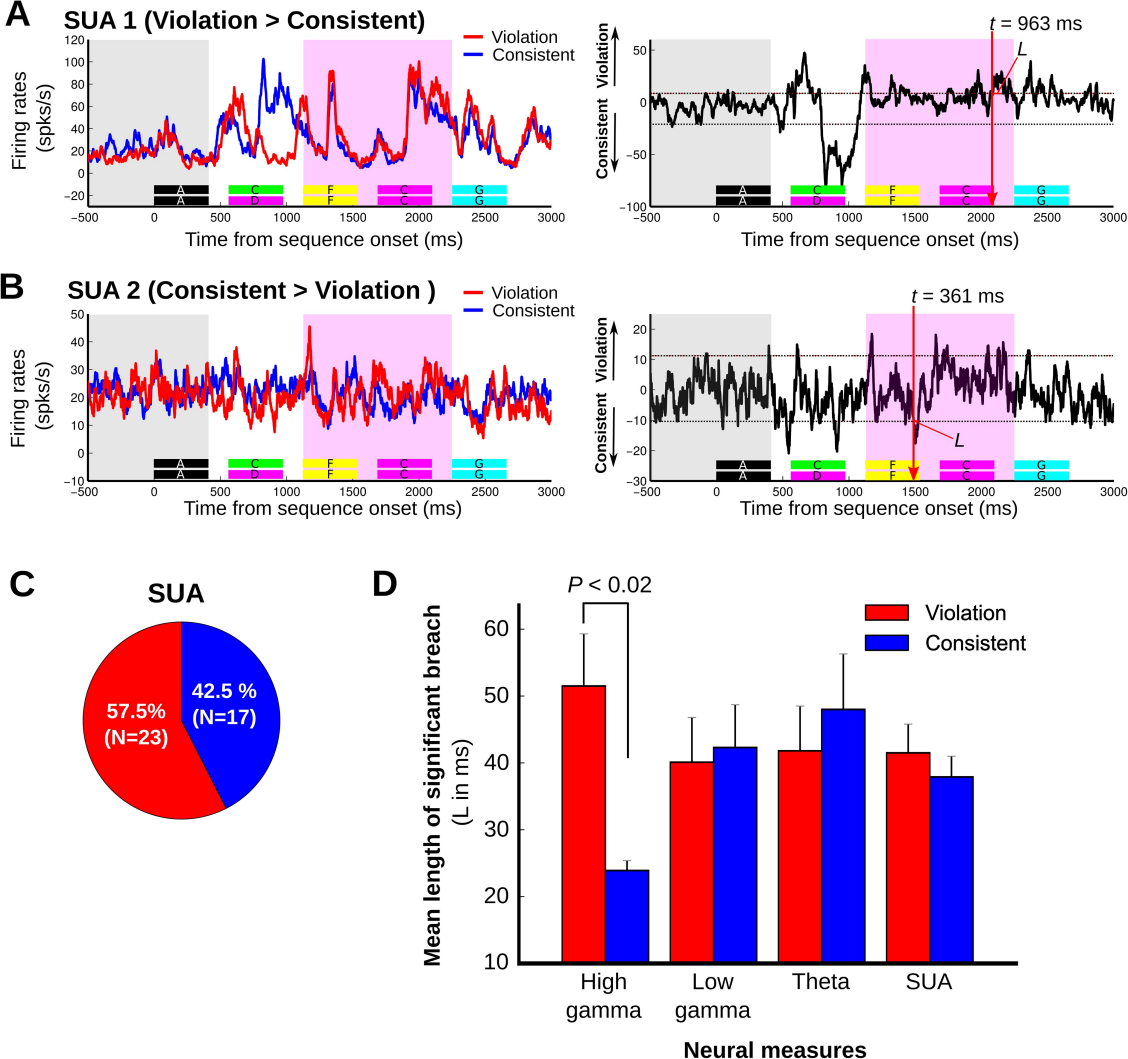
Exemplary monkey neuronal (single-unit activity [SUA]) response sensitivity to the sequencing context. Figure format is the same as in Fig 4. A. Exemplary SUA showing significant response sensitivity to the violation sequence with a sensitivity latency of 963 ms (*L* = 77 ms). B. Exemplary SUA showing significant response sensitivity to the consistent sequence with a sensitivity latency at 361 ms (*L* = 36 ms). Note that peak differences in panels A–B occurring before these reported significant latencies were either not significant by the joint criterion of response magnitude and duration (Materials and methods) or occurred before the start of the analysis window and were thus not included for analysis. C. Proportions of neuronal responses to sequences showing significant effects in response to either the consistent or violation conditions. D. Magnitude of the context-dependent response to violation sequences (red) and consistent sequences (blue), measured as the length of time above the significance threshold (*L*) across different neural response measures: high-gamma (*n* = 40), low-gamma (*n* = 40), and theta (*n* = 36) local field potential (LFP) power, including SUA (*n* = 40). High gamma showed a significantly greater response magnitude to the violation sequencing context (*p* < 0.02). The vertical lines denote the standard deviation.

The number of recording contacts in humans is limited, and access to single units was not possible; thus, using monkeys as a model system, with a more substantial sampling of population and single neuronal responses, we conducted further analyses that link the fundamental scale of neuronal processing in the brain to results obtained at the other neural scales (i.e., local field potentials and oscillatory coupling; Fig 4 and Fig 5). Analysis of local field potential and single-unit responses confirmed the relatively late auditory neuronal sensitivities to the sequencing context seen with oscillatory coupling responses (>600 ms), and the results substantiated the observation of different subpopulations of responses sensitive to either the consistent or violating ordering relationships. Across the 145 LFP sites, the proportion of responses to the sequencing context (differential violation versus consistent responses during the acoustically matched sequence pairs in the probe stimulus analysis window) were significantly different for high gamma (violation versus consistent, χ^2^ = 4.9, *p* < 0.03, *n* = 36) but not for theta and low gamma oscillations (theta: χ^2^ = 0.44, *p* > 0.50, *n* = 36; low gamma: χ^2^ = 0.1, *p* > 0.70). This is seen as a relatively even split in theta and low-gamma LFP power (Fig 4C) and SUA (Fig 5C) in response to the violation or consistent sequences, with high-gamma responses tending to be more prominent in response to the violation sequencing context.

Next, we analyzed the strength of the sequencing context effect in the LFP and SUA data in terms of their magnitude and duration (Fig 4 and Fig 5). This analysis showed that all neuronal LFP power and SUA responses to the sequencing context had substantial breach durations (~40 ms) above the significance threshold (mean ± standard error of the mean [SEM]; high gamma, 42 ± 5.7 ms, *n* = 40; low gamma, 41 ± 4.6 ms, *n* = 40; theta, 45 ± 5.2 ms, *n* = 36; SUA, 40 ± 2.8 ms, *n* = 40). High-gamma power, in particular, showed a significantly greater average response magnitude to the violation context than to the consistent sequencing context (Fig 5D; 52 ± 7.8 ms versus 24 ± 1.5 ms, violation versus consistent, *p* < 0.02, Wilcoxon rank-sum test). The observed sequencing context effect appears to be independent of the differential response to the sounds prior to the probe stimulus window, since there was no significant correlation between the magnitude of the high-gamma response to the sound preceding the violation transition and the magnitude of the context-sensitive response during the probe stimulus window (*r* = 0.14, *p* = 0.54, Pearson correlation; see S3 Text and S8 Fig).

**Fig 6:**
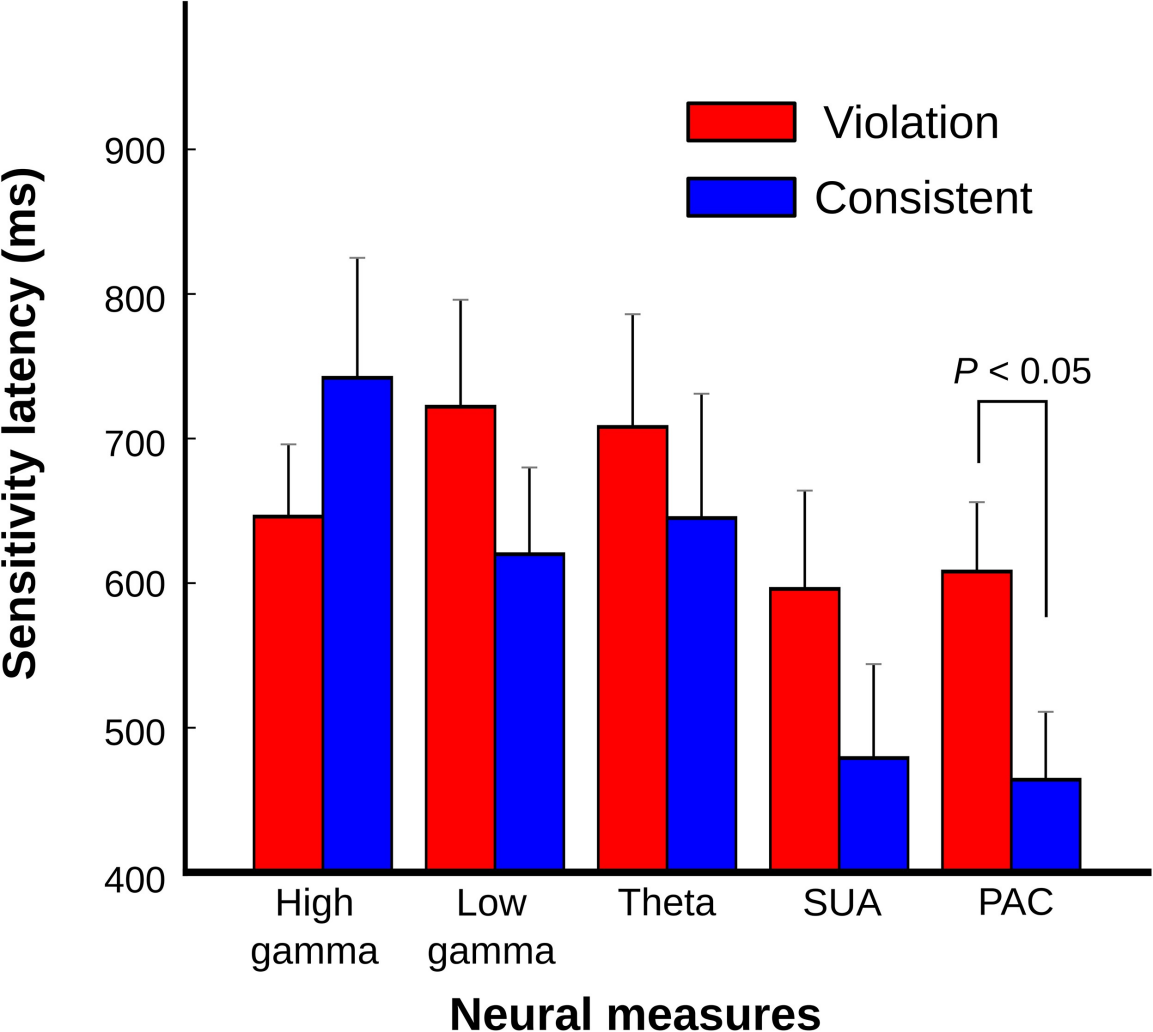
Sequencing context sensitivity latencies across neural response measures. Mean sensitivity latencies in response to violation (red) and consistent sequences (blue) shown for different neural response measures (high gamma, low gamma, theta, single-unit activity [SUA], and phase–amplitude coupling [PAC]). The neural population data for each type are the same as those used for Fig 4C, Fig 5C and 5D. The different neural response measures showed relatively late sensitivity latencies (high gamma, mean ± SEM: 677 ± 43 ms, *n* = 40; low gamma: 674 ± 48 ms, *n* = 40; theta: 680 ± 57 ms, *n* = 36; SUA: 547 ± 48 ms, *n* = 40, PAC: 538 ± 34 ms, *n* = 85, Fig 6) with significantly shorter latencies in response to consistent sequences compared to violation sequences in the oscillatory coupling response: PAC (violation: 607 ± 48 ms, *n* = 44; consistent: 463 ± 47 ms, *n* = 41; Wilcoxon rank-sum test, *p* < 0.05).

To evaluate whether the various neural response measures differed in the latency of their responses to the sequencing context (Fig 6), we used an ANOVA with the response latencies as the dependent variable and the factors monkey, sequencing context, and type of neuronal response (PAC, LFP across three frequency bands, and SUA). The analysis revealed a main effect of neuronal response type (*F*_*4*, *221*_ = 2.776, *p* < 0.03) with no interactions (all *p* > 0.1). Post-hoc comparisons showed that PAC response latencies were comparable to those in the SUA but were significantly shorter than responses based purely on LFP power (Fig 6; Fisher’s least significant difference [LSD], *p* < 0.03). Moreover, the PAC sensitivity latencies were significantly later in response to violation sequences than to consistent sequences (Fig 6).

## Discussion

This study identifies the auditory cortical neural signatures associated with monitoring learned sequence ordering relationships. The oscillatory coupling in response to the nonsense words and sequence ordering relationships are seen to be remarkably similar in humans and monkeys, and these results are further informed by local field potential and single-neuron responses in the monkeys as a model system. After exposing humans and monkeys to rule-based, nonsense word sequences, we demonstrated the following: (i) transient low-frequency neural oscillatory phase couples with high-gamma band amplitude in response to the nonsense word sequences in the auditory cortex, which was robust even with the stimulus-evoked response removed; (ii) the previously experienced temporal ordering relationships modulate neuronal responses and oscillatory coupling after a violation transition (~450–700 ms); (iii) oscillatory coupling effects occurred in tandem with spiking activity (single neuron) responses and led effects in local field potential power; and (iv) neural responses to the sequence ordering relationships were time sensitive, with, for example, oscillatory coupling responses occurring early in time to consistent sequencing relationships prior to further modulations of oscillatory coupling in response to violations of the sequence ordering relationships.

### Evolutionarily conserved nested oscillatory coupling

The results appear to address any remaining uncertainty regarding whether human nested coupling in response to complex sounds, such as speech, is at all like the auditory cortical coupling elicited by the same type of stimuli in other animals. General cross-species correspondences between neuronal oscillations in rodents, monkeys, and humans have been noted (e.g., [23]) and are taken as evidence of broadly evolutionarily conserved oscillatory processes and mechanisms. A prominent neurophysiological model of auditory cortical segmentation of speech [7, 9, 26] postulates that auditory cortical oscillations are entrained by the multi-timescale structure in speech sounds (e.g., phonemic, syllabic). Speech syllables entrain low-frequency theta oscillations (4–8 Hz), which in turn couple with high-frequency (>30 Hz) gamma amplitude, regulating neuronal excitability and segmenting speech into an appropriate temporal granularity. Moreover, studies in nonhuman animals have reported auditory cortex activity that is phase-locked to the temporal envelope of complex sounds, including vocalizations [10, 32], speech syllables [33], and natural sounds [11, 34], although not always at theta-band frequencies. However, the lack of direct comparisons between humans and nonhuman animals has left the comparisons and relationships to single-neuron responses tentative and the issue unresolved. Our results show that nested neuronal coupling to nonsense words is remarkably similar across the species, and effects occur in tandem with single-unit responses. These cross-species observations raise the important question of what are the neurobiological processes supporting human speech perception, if complex sound segmentation is seen to be a general property of neural processing within and around the primary auditory cortex. Part of the answer might be that downstream processes, such as those in the human superior temporal gyrus and sulcus, support more speech perception–specific representations [35].

Since speech production and perception are unique to humans, it is unlikely that nonhuman primates perceived the nonsense words differently to other complex natural sounds. With training, monkeys and rodents can learn to discriminate speech [36, 37]. For our paradigm, it is critical that the monkeys and humans are able to perceptually distinguish the different nonsense words in order to recognize the ordering relationships [19, 21]. However, because of their experience with speech and language, humans might process the phonotactic content (and other aspects of speech) contained within the nonsense words differently than would monkeys. Nonetheless, given these and other unavoidable differences in testing the two species or how they might perceive the stimuli, the similarities in the form of nested coupling seen in the human and monkey auditory cortex are all the more remarkable.

Our human oscillatory coupling results in response to speech sounds are generally consistent with those from the initial report of theta–gamma coupling in human intracranial recordings [25]. The prior study used word recognition tasks, leaving uncertain the extent to which sublexical stimulus features are sufficient to evoke theta–gamma coupling. Accumulating evidence from this and other studies support the notion that theta signal modulation and theta–gamma coupling can arise as a function of speech-related processing [38–40], which, as we show, is evident in human and monkey neuronal oscillations within the auditory cortex even with stimulus-driven responses removed from analysis. These oscillations are further modulated by learning and mnemonic operations, as we now consider.

### Sequencing relationships modulate hierarchically nested oscillations and neuronal responses

By design, we used violations of specific sequencing relationships to ensure that the subsequent probe stimulus period that was analyzed across the “violation” and “consistent” sequence pairs contained the same acoustical elements that were being compared across the conditions. Thus, the contribution of pure stimulus entrainment in the observed PAC should be equal across the conditions. Note also that the wavelet filtering across the two sequence conditions was identical. Our results also confirmed that initial stimulus-driven, pure power or phase effects cannot easily explain the results, since we removed the trial-by-trial evoked stimulus–response from the LFP signals (see S3 Fig, Materials and methods). Moreover, to rule out potential artificial PAC coupling, which can occur for a number of reasons [41], we used phase-clustering correction [42] and trial-shuffled permutation testing before calculating PAC. Beyond the PAC elicited by the nonsense words, we observed that PAC was sensitive to the sequence learning context, seen as dynamic modulation of the PAC for a substantial amount of time after a violation transition (~450–700 ms over the next two sounds in the sequence), which can occur independently of any stimulus time-locked phase coherence (S3 Fig). Therefore, a parsimonious general explanation of the results is that non-stimulus–driven effects are responsible for the observed PAC of neuronal oscillations in both the human and monkey auditory cortex. The analyses in relation to SUA responses suggest that oscillatory coupling occurs in tandem with single neuronal responses, with effects on populations of local neurons’ (i.e., time-averaged LFP gamma power) activity following (Fig 6).

The between-word transitions in our sequences occurred at a regular 1.8 Hz rate. Thus, one prediction is that monitoring the sequencing transitions would entrain low-frequency delta oscillations and affect the form of nested oscillations to more strongly involve a delta component [6, 29, 39, 43–46]. Although our analysis could only measure as low as 3 Hz, the observed PAC effects are clearly not specific to delta (<4 Hz), however, involving also theta (4–8 Hz) phase coupling with gamma amplitude (S1 Text and S4 Fig). Sequence learning effects on theta, in particular, at least on the surface appear to resemble those shown for other learning or mnemonic processes. For instance, theta oscillations in the auditory cortex measured by magnetoencephalography (MEG) are modulated during speech recognition when speech is comprehensible [38]. Also, when participants monitor syntactic properties in sentences and store certain relationships in memory, electroencephalography (EEG)-based theta frequencies are modulated in frontoparietal brain regions [28].

Some of the neuronal effects related to sequence learning reported here could be intrinsic to the auditory cortex, given that rapid, learning-related, receptive field plasticity occurs in the auditory cortex [47]. It is also possible that the auditory cortex interacts with a broader network of regions, and we consider some likely candidate regions and physiological processes involved. For example, it is broadly accepted that the hippocampus is important for encoding and retrieval during explicit forms of learning and memory [13, 48, 49]. Recent evidence using implicit (statistical or associative) learning tasks has raised the interesting possibility that the hippocampus is also involved in more implicit forms of learning, assisting in evaluating previously learned associations in relation to novel incoming input [50–53]. The hippocampus also has intrinsic neuronal oscillations in the theta-frequency band that can be modulated by learning and memory [54–61]. Thus, hippocampal neuronal oscillations seem to be important for sampling temporally discrete sequential events [13], processes that are modulated by cognitive operations.

Much less is known about the role of the sensory cortex, especially with regards to how the monitoring of previously experienced sequencing relationships impact on neuronal oscillations and coupling. Recordings of auditory neuronal spiking responses in songbirds using a statistical learning paradigm showed stronger responses to novel low-probability transitions versus familiar sequences [62]. In the monkey inferior temporal cortex, neurons adapt their responses to an image whose presence is predicted by a leading image [63], and the neurons respond more strongly to the image not predicted by the preceding image(s) [64]. However, a study in rodents using a fixed A–B–C–D sequence of visual gratings at different orientations showed strong potentiation in response to the familiar predictable sequence in the rat primary visual cortex in relation to sequences with unpredictable transitions between the stimuli [65]. Our work could help to reconcile and extend some of these seemingly discrepant findings across brain regions and species: we see similar neuronal responses and oscillatory signatures in humans and macaques associated with previously experienced sequence ordering relationships that are seen prominently, but not exclusively, for violations of sequencing relationships (Fig 4 and Fig 5).

The relatively late sensitivity to the sequencing relationships is an interesting observation, since it is generally consistent with the approximate time of behavioral eye-tracking sensitivity in response to specific violations to these sorts of sequencing relationships (~600 ms) [19]. Also, using the same stimuli and paradigm as the ones used here, monkey EEG results showed a late event-related positivity at ~500 ms ([66], also see [67]). Thus, our results establish the primate auditory cortex as an important site for monitoring previously experienced sequence ordering relationships, whereby specific violations to the ordering relationships modulate nested coupling at a time when the effects appear to be behaviorally meaningful and seem to elicit large-scale event-related potentials. However, structured sequence learning is related to but not equivalent to grammatical violations in natural language, which in humans are known to elicit late effects in EEG potentials such as the P600 [68, 69]. In all cases, our study shows an approach for identifying the auditory cortical correlates of certain EEG or MEG responses, some of which might have hierarchically nested coupling as an underlying mechanism. In humans these processes can be compared with natural language operations.

The auditory cortical effects that we observe occur later than other types of contextual auditory cortical effects [70], raising the question of which brain regions are interacting with the sensory cortex to elicit these learning-related effects. The hippocampus is a good candidate, as we considered above, and neural processes in this region also show relatively late sensitivity to violations of other types of sequence ordering relationships (300–600 ms) [71]. Additionally, we predict that the ventral frontal cortex plays a key role in monitoring sequence order for previously learned relationships [72]. Namely, a recent comparative functional magnetic resonance imaging (fMRI) study in monkeys and humans using the same paradigm showed that the ventral frontal and opercular cortex is consistently engaged across participants and in both species in response to violations of the ordering relationships [20]. However, possibly because of the mixed subpopulations of different types of neuronal responses that the current study identified in the auditory cortex, the involvement of the auditory cortex was not clear in the prior comparative fMRI study. This was the case even with the fMRI data reanalyzed here, focusing specifically on the human and monkey auditory cortex (S4 Text and S9 Fig).

Our monkey neurophysiological data provided more systematic coverage of SUA and LFP activity in the auditory cortex than was possible with the human neural recordings, revealing a dichotomy of neuronal response types. These combined observations are difficult to explain by early stimulus-specific adaptation (SSA) because of the rather late occurrence of the effects that we see (~600 ms), but see the long-lasting effects reported by Yaron and colleagues [73]. Also, rapid SSA effects would elicit a reduction in neuronal responses to stimulus regularities [74, 75], whereas we also see stronger responses to the legal (consistent) versus violation sequences in a subpopulation of neurons. We also find evidence for different types of neuronal responses across scales (Fig 4C), with mainly high gamma showing significantly greater magnitude and proportions of responses to the violation sequencing context (Fig 4C and Fig 5D). These aspects of the results appear to be more comparable to the potentiation of neuronal responses to predictable stimulus transitions, as seen in the rodent primary visual cortex [65], although, again, it is worth noting that the effects here occur well after stimulus onset.

**Fig 7:**
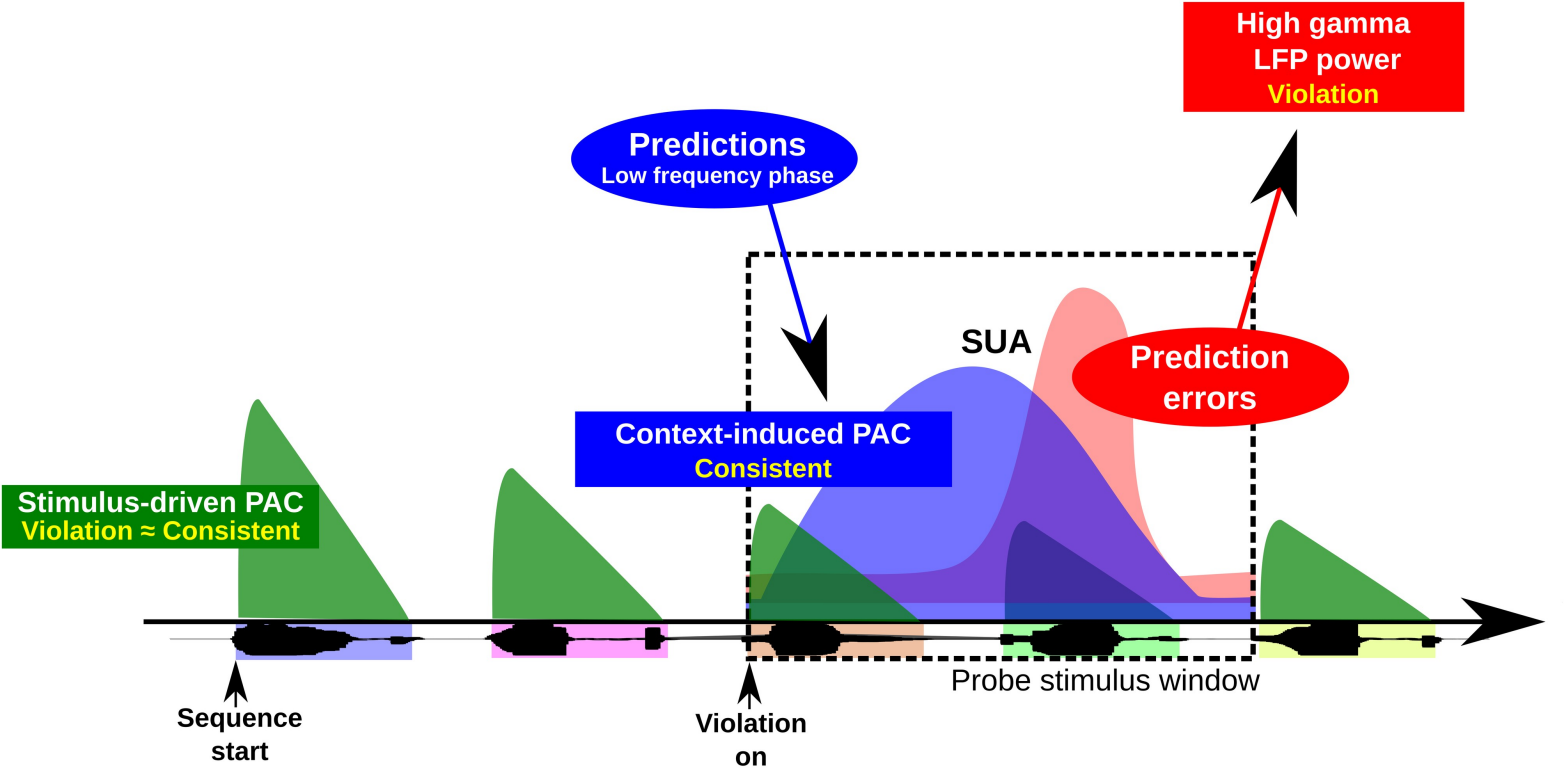
A physiological model of sequencing predictions in time. Sound onset is known to coordinate low-frequency phase and high-frequency amplitude neural oscillations (see text). Less is known about how context-dependent sequence learning effects modulate neural oscillations and coordinate with neuronal responses. This physiological model based on the results of the study shows that after exposure to structured sequencing relationships, different neural signals (local field potential [LFP], single-unit activity [SUA], oscillatory coupling) show context-dependent response modulation that can lag responses due to sound onset. Prediction signals, reflected in phase–amplitude coupling (PAC) and possibly emanating from hierarchically higher brain areas, occur throughout the sequences when the ordering relationships are consistent with the learned sequence ordering relationships (note that for simplicity this is only illustrated in the black dashed line box for one violation transition). This prediction signal is modulated later in time when a sequencing violation occurs (a prediction error), also affecting local neural processes (see results in Figs 5 and 6).

The predictive coding theory of brain function can potentially explain and be informed by our results. Predictive coding models of perception posit that low-frequency prediction signals from hierarchically higher areas are compared with incoming sensory information from hierarchically lower areas, generating high-frequency error signals in the form of gamma oscillations that are fed forward to higher areas [76–79]. Our results on oscillatory coupling and the observation of a more prominent high-gamma signal in response to violation sequences (Fig 5) are generally consistent with these models. Innovation is provided here by the observation of fundamental interactions between low- and high-frequency oscillations as a function of learned sequencing relationships likely encoded via intrinsic auditory cortical interactions with other areas of the brain (Fig 7). Such interactions are a possible manifestation of the gamma error signal from a lower-frequency (possibly beta band) prediction input [77, 78]. Interestingly, theta and/or alpha oscillations have also recently been shown to relate in magnitude to the precision of higher predictions [79] and as a feed-forward process in the primate visual pathway [78, 80], indicating wider roles for these low-frequency oscillations than acknowledged in current theory. Enhanced low-frequency oscillations may therefore be important for strengthening the gamma prediction error signal for learned sequencing operations on sensory input. Moreover, informing predictive coding models, we discovered a candidate physical mechanism for comparing ongoing prediction signals with incoming sensory information, based on PAC that is delayed beyond the PAC directly resulting from the signal itself. This is illustrated in Fig 7, whereby prediction signals, induced here by the processing of consistent sequencing relationships, lag those associated with stimulus onset. When there is a prediction error (in our case, a response to a sequencing order violation) there is a further modulation of PAC and local neuronal processing. Future studies could seek to establish the feed-forward and feedback interactions that may occur between the sensory cortex and other brain regions, including specifying the cortical layers where the reported effects occur [81, 82]. The work here provides the framework for such pursuits that can now strongly benefit from studies in animal models to complement and extend the observations that can be obtained from intracranial recordings in humans.

In summary, the findings support the notion that hierarchically nested oscillations, such as theta–gamma coupling in response to complex sounds, reflect general auditory physiological processes in primates. Moreover, the human and monkey auditory cortex show the neural signatures of learning- or experience-based sequence monitoring in the form of modulations of transient, low-frequency (including theta) oscillations coupling with gamma and concomitant effects in single neurons and populations of neurons at specific times as a function of the predictability of the sequence ordering relationships. Overall the results imply that these processes are unlikely to have evolved, at least at this level, in a uniquely specialized manner in humans. The described physiological processes inform neurobiological research on the neural bases of learning and predictive coding. The work opens the door for more systematic study of the mechanisms for these processes in animal models with more direct comparisons to neural processes in humans.

## Materials and methods

### Ethics statement

All macaque procedures performed in this study were approved by the UK Home Office (project license: 70/8318) and by the Animal Welfare and Ethical Review Body at Newcastle University. The work complies with the Animal Scientific Procedures Act (1986) on the care and use of animals in research and with the European Directive on the protection of animals used in research (2010/63/EU). We support the principles of the consortium on Animal Research Reporting of In Vivo Experiments (ARRIVE).

All human research procedures were approved by the University of Iowa Institutional Review Board (IRB ID #: 200112047). Prior informed written consent was obtained from each subject participating in the study. These guidelines are also consistent with European Ethical Guidelines (Helsinki Declaration and H2020 EU guidelines).

### Monkey experiments

Two adult male rhesus macaques (*Macaca mulatta*, weighing 9 and 13 kg and 5 and 8 y old, respectively) from a group-housed colony were studied. The pen sizes in our colony range from 130 × 240 cm to 215 × 240 cm width and depth with 230 cm height. Hatches between neighboring pens are used to increase the space available to the animals.

#### Task and stimuli

Nonsense word sounds were used within sequences that were consistent with the allowed AG transitions between the nonsense words or violated them in very specific ways. The transition graph (Fig 1B) consists of multiple forward-branching relationships and has considerable variability in the transitional probabilities [19, 21, 30]. The paradigm consists of five nonsense words serving as elements to the sequences (A, C, D, F, and G). The words were “yag,” “lek,” “kem,” “pav,” and “rop” (Fig 1A). The nonsense words were produced by a female human talker, digitally recorded with an Edirol R-09HR sound recorder (Roland Corp.) in a sound-attenuating acoustical chamber. The mean duration of the nonsense words was 413.6 ms (yag = 413.7 ms; kem = 413.1 ms; lek = 414.0 ms; pav = 413.7 ms; rop = 413.5 ms). The amplitude of each nonsense word was root mean square (RMS) balanced. The stimulus presentation rate was 1.8 Hz per nonsense word with an interelement interval of 150 ms. The consistent and violation sequences were balanced in duration and other acoustical aspects: for details, see [19, 66]. Sound waveforms were amplified, attenuated (PA4, TDT), and delivered through audio speakers at ~75 dB SPL. The sound level was calibrated at the animal's head position using an XL2 sound level meter (NTI Audio) before every recording session. The electrophysiological recording sessions were conducted in a darkened, foam-lined, single-walled acoustical chamber (IAC). The animals had previously been acclimated to periods of head immobilization using operant training with positive reinforcement. The macaque was seated in a recording chair with the head immobilized, facing a video monitor approximately 60 cm in front of the subject (24” Samsung, LCD). Two free-field speakers (Creative Inspire T10) were horizontally positioned at approximately 30° visual angle.

Each session consisted of a 30-min exposure phase in which the animals passively listened to the habituation sequences. During the exposure phase, a set of exposure sequences that followed the ordering relationships specified by Fig 1B were presented in random order (all the sequences used in this study are shown in S1 Fig). This exposure phase was followed by a 30-min testing phase (~260 trials). During the testing phase, a randomly chosen sequence of the eight “consistent” or “violation” testing sequences was individually presented over the audio speakers while the animals fixated on a spot on the video monitor.

The animals had previously been trained to perform a fixation task during sound stimulation, which was assessed using an infrared eye tracker (Arrington Research, AZ, US). During the testing phase, they initiated an experimental trial by fixating on a spot at the center of the monitor for 500 ms, triggering the presentation of a randomly selected testing sequence from the eight testing sequences available (S1 Fig). The macaque was required to fixate on the spot for 3,000 ms (within a ~2–4°-diameter fixation acceptance window centered on the spot) until the end of the testing sound sequence presentation to obtain a juice reward. They received the juice reward at the offset of the fixation spot for correctly fixating during testing sequence presentation. Reward delivery occurred between 50 to 67 ms after the end of the sequence presentation. The fixation task minimized eye movements during sequence presentation, and only successfully completed fixation trials (>90% correct performance) were analyzed. The task was self-paced and intersequence intervals were on average 7.6 ± 2.3 s. Typically, the animals quickly engaged the fixation spot to start the next trial or took a brief break before starting the next trial.

#### Monkey electrophysiological recordings

MRI was used for guiding chamber placement and the electrodes. The MRI structural and functional data were obtained with a 4.7T scanner (Bruker BioSpin, Etlingen, Germany). A customized, MRI-compatible head post and cylindrical recording chamber (19 mm diameter, PEEK) were implanted under aseptic conditions during general anesthesia. The recording chamber was positioned stereotaxically over the right hemispheres of both animals to target the caudal, tonotopically organized auditory cortex (see S1 Fig and S6 Fig; [83, 84]). The locations of the chambers of both animals were later also physiologically confirmed using the topography of tonotopic responses of SUA, covering the caudal core (including field A1) and the lateral belt of auditory cortex (S1 Fig). Multiple independently driven tungsten microelectrodes (~1.0 MΩ, epoxylite insulation, FHC, Bowdoin, ME) were used for the extracellular electrophysiological recordings. Guide tubes were first advanced through the dura to protect the electrode tips and to prevent electrode deflection. After the guide tubes were in place, the electrodes were independently advanced using a remote-controlled, multichannel microdrive system (NAN-SYS-4; Plexon. Inc., Dallas, TX).

Search stimuli (including tones, noise, and complex sounds) were used to ensure that recordings were from the auditory cortex. However, the recorded locations, neuronal spiking activity, and LFPs were not selected by their stimulus response preference nor the shape of the waveforms of the spiking activity. The search stimuli were only used to confirm a significant auditory response to any of the sounds. The neuronal signal from each electrode was sent through a head stage (gain one, HST/8o50-G1, Plexon Inc.) and then split into spiking and LFP activity through a preamplifier (PBX2/16sp/16fp, Plexon Inc.). The spike signals were bandpass filtered between 150 and 8,000 Hz, further amplified, and then digitized at 40 kHz. The LFP signals were filtered between 0.7 and 500 Hz, amplified, and digitized at 1 kHz. During the electrophysiological recording session, spiking activity was sorted using voltage thresholding and then a template-matching principal component analysis (PCA) clustering method (RASPUTIN, Plexon). The frequency tuning profiles of the neuronal responses were also estimated during the experiment (Neuroexplorer, Nex Technologies, MA). Throughout the recording sessions, we also monitored spiking activity visually with an oscilloscope (HM407-2, HAMEG) and aurally through headphones (HD 280 Professional, Sennheiser). Since the signal on each electrode often contained activity from more than one SUA, offline we separated the multiunit spike trains into single-unit spike trains using PCA (Offline Sorter, Plexon, Inc). The degree of separation of multiple clusters was inspected using MANOVA when more than one cluster was identified (i.e., multiunit spike trains). We applied a threshold of *p* < 0.01 to identify whether the observed clusters recorded from the same electrodes came from a separate SUA, and only well-isolated units were included for further analysis. The temporal stability of SUA was inspected using the PCA analysis, and we excluded data that was temporally discontinuous. The interspike interval was also inspected to better ensure that results were from separate single units, ensuring that the interspike interval was greater than the neuronal refractory period (1–2 ms).

The timing of behavioral events, reward, and stimuli were controlled by a Windows CORTEX (Salk Institute) dual-computer system through a 12-bit D/A converter (CIO-DAS1602/12, CIO-DIO24, National Instruments). Continuous data such as audio waveforms and eye traces measured by the eye-tracking system were sent to the Plexon MAP data acquisition system (Plexon, Inc.) and stored with the spiking activity and LFP data.

#### Spike density function: Monkey SUA

For the monkey SUA data, a spike density function was created to construct spike density peristimulus time histograms (PSTHs). This involved convolving spike counts with an asymmetric exponential function (time constants for the growth function: phase = 1 ms, decay phase = 20 ms) [85]. This asymmetric procedure avoids the influence of spiking activity during the prestimulus period, as would result from a symmetric kernel (e.g., Gaussian kernel), and improves the precision in measuring response latencies.

#### Recording sites and tonotopic maps: Monkey SUA

The neuronal tonotopic response maps (S1 Fig) in combination with the monkeys’ fMRI tonotopic maps show that the recordings encompassed the high- to low-frequency regions of A1 in M1 and M2, extending partially into R in M2 and including part of the lateral belt regions adjacent to these fields. To construct tonotopic maps of neuronal best-frequency responses for each animal, we first normalized the mean firing rates of neuronal spiking responses by subtracting the average baseline firing rate (300 ms period prior to sound onset) across all of the pure tone stimuli. Seven pure tones were used for stimulation, ranging from 220 to 14,080 Hz in octave steps, and/or data was obtained using ten pure tones ranging from 32 to 16,384 Hz in octave steps. The tones were randomly presented and the data were obtained before or after the experimental recording session. Tuning curves were constructed for auditory neurons with significant increase in their firing rates (>3 SD) in relation to the baseline period. For such neurons, we defined a best-frequency (BF) response as the tone frequency eliciting the maximal response during the 300-ms pure tone presentation period across the tone frequency range. For this study, we did not attempt to separate auditory core and belt recordings, which would require additional data using other sound stimulation conditions. We used this approach primarily to confirm recordings that occurred from the tonotopically organized macaque auditory cortex.

#### Monkey LFP (power) and SUA analyses

We evaluated whether the LFP and spiking (SUA) responses in the monkey auditory cortex could be modulated by the artificial grammar (AG) context (contrast between responses to consistent versus violation sequences or vice versa). The difference waveform plots (Fig 4 and Fig 5) were created by subtracting the grand average response to the consistent sequence from the grand average response to the corresponding violation sequence (violation–consistent). The start of the analysis window for the LFP power-based responses was shifted to 150 ms after the onset of the violation transition to remove any aftereffects of preceding responses (Fig 4).

To quantitatively evaluate whether the activity in response to the same acoustical elements was different depending on whether a violation or no-violation transition preceded the response to the acoustical elements, a difference waveform was calculated from the grand average responses across all trials. This was done separately for both LFPs and SUA. Then, a difference response waveform confidence interval was defined (99% or 1% confidence interval) using a permutation procedure defining a null distribution of difference waveforms computed from a baseline period. The baseline period used for this analysis consisted of responses during the silent period prior to the onset of the sequence and the first acoustical element “A” that was present as a starting element in all sequences (i.e., a baseline 913-ms window including the 500 ms prior to the sequence onset throughout the end of the first element “A”). Furthermore, for breaches across the confidence interval (CI), we also calculated the discrete duration in time (“*L*”) above the significance threshold, which was statistically defined using two permutation tests (*p* < 0.05) on the magnitude and the duration of the breaches above the CI in either direction (preference for violation or consistent sequence). LFP or SUA responses that showed a significant breach in magnitude and duration across the CI were identified as showing a significant sequence condition latency effect (“sensitivity latency”). We ensured that the calculation of sequencing context sensitivity latency started only after any preceding response difference became not significant (see S8 Fig and S3 Text).

### Human experiments

#### Human experiments

Three adult neurosurgical patients participated in this study (two males [H1, H3] and one female [H2]; ages 29, 31, and 37). All three subjects were right handed (+100 RH lateralization index). H1 (L307) and H3 (L357) showed left-dominance language lateralization by a Wada test [86]. H2 (R316) had no indication of atypical language organization and had suspected right hemisphere dysfunction, thus the Wada test was not performed. Audiometric and neuropsychological evaluations were performed on all of the subjects before the study. H1 was noted to have a naming deficit and a hearing deficit in the right ear (a notch in their audiogram of 40 dB HL at 4 kHz). H2 was noted to have some cognitive deficits in spatial skills and visual memory with no hearing deficits. H3 was noted to have a modest verbal memory deficit and no hearing deficits. All of the participants were native English speakers.

#### Patients, electrode implantation, and intracranial recordings

The HG was not found to be involved in the generation of epileptic activity in any of the human patients reported here. The methods for human intracranial electrophysiological recordings are similar to those reported elsewhere [87]. Briefly, eight contact (70–300 kΩ) depth electrodes were implanted along the axis of HG in one hemisphere (on the left hemisphere for H1 and H3 and the right hemisphere for H2). Whole-brain MRI and CT scans were performed before electrode implantation for each subject. The electrode positions were determined using postoperative MRI scans by coregistering the electrode locations with the subject's preoperative, high-resolution, T1-weighted structural MRIs (0.78 × 0.78 × 1.0 mm). Then, the MRI with the electrode locations was 3-D rendered. The boundaries in the two subjects between the posteromedial and the anterolateral aspects of Heschl’s gyrus (S5 Fig) are defined using the morphology of the short-latency auditory-evoked potentials (AEP) to sound click trains and frequency following responses [88].

#### Task and stimuli in the human study

Both exposure and testing sequences used in the human experiment were identical to the ones used in the monkey experiment shown in S1 Fig. However, the 8 testing sequences were tested in one testing session for the human experiment rather than in two testing blocks as with the monkeys. The duration of the exposure session was 10 min, and the testing session lasted 14 min. The exposure session was conducted first, followed by the testing session. In the exposure session, 8 sequences were randomly repeated 20 times (a total of 160 sequences) with sequence onset asynchrony of 5.1 sec. In the subsequent testing session, the 8 consistent and 8 violation sequences were randomly presented ten times each (a total of 160 trials). The subject was comfortably sitting on the bed and was only required to listen passively to the stimuli. The sound sequences were delivered from two free-field speakers located 1 m away from the subject's head on both sides of the bed. Stimulus presentation was controlled by Presentation software (www.neurobs.com). LFP data and sound waveforms were recorded through a TDT RZ2 processor, in which signals were amplified, filtered (0.7–800 Hz bandpass, 12 dB/octave roll off), digitized at 2,034.5 Hz, and stored for subsequent offline analysis.

H1 agreed to participate in the behavioral follow-up to the intracranial recordings experiment 61 d after their surgical monitoring period (S2 Fig). This allowed us to evaluate whether the subject could behaviorally differentiate between consistent and violation sequences using the same sequences used in the electrophysiological experiment. For comparative macaque and typical human sequence learning behavioral data on this task, see [2, 3]. The identical sound sequences used in the electrophysiological experiments were used. First there was an exposure session, followed by a testing session. In the exposure session, eight sequences were randomly repeated seven times (a total of 56 trials), and in the testing session, eight consistent and eight violation sequences were randomly presented five times (a total of 80 trials). During the testing phase, the subject heard one of the testing sequences randomly selected for each trial and was required to respond by using two buttons to classify the test sequence heard as either following the “same” ordering pattern as the exposure sequences previously heard (a consistent sequence) or following a “different” ordering pattern (violation). A forced-choice procedure was used in which the next trial only began once a response was given. No feedback was given to the subject for their behavioral responses to the testing sequences. We conducted two repeats of the exposure and testing sessions, with the whole experiment taking ~25 min to complete. Reaction times after sound sequence offset were compared between the consistent and violation conditions for correct response trials using a Mann–Whitney rank test (S2 Fig).

### Data analysis: Human and monkey experiments

#### Data preprocessing

Data selection, pre-processing, and data analyses were conducted using MATLAB 7.1 or Python 2.7. For monkey LFPs, the 50-Hz electrical line noise was removed using multitaper time-frequency decompositions with a 1-s window and 0.5-s steps (Chronux package function, rmlinesmovingwinc.m, http://chronux.org/). A time bandwidth product of five and nine Slepian taper functions was used.

For human LFP recordings, the data were first downsampled to 1 kHz. Large-amplitude timetransients, identified by thresholding, were suppressed by multiplying the signal with a 0.2-s Hann-window notch centered at the time of the transient. To minimize the potential for spectral leakage artifacts and resultant cross-frequency contamination, line noise was removed according to an adaptive filter procedure using a complex, demodulation-based, time–frequency decomposition [89, 90].

For all human and monkey comparisons, the neural responses to violation and consistent sequences were analyzed using a probe stimulus analysis window comprising two elements and two interstimulus intervals (1,126 ms) after a violation had occurred in the violation sequence and the corresponding acoustical elements in the matching consistent sequence (see “probe stimulus analysis window” in Fig 1C and S1 Fig). Thus, the difference of neural results in response to the two sequences being compared cannot arise from acoustical differences or filtering artefacts.

#### Time-frequency analysis

To extract estimates of band-specific, time-varying phase and power in the LFP data, a complex Morlet wavelet convolution was used (sinusoid windowed with a Gaussian, three wavelet cycles). The ERSP [31] was calculated for each frequency and time point. ERSP measures event-related changes in the power spectrum using a sliding window that is averaged across trials. The consistency of phase angles across trials (ITC) was calculated by the length of the average population vector of unit-length vectors from each trial. The length of the average vector reflects the proximity of vectors across trials. It is calculated using Euler's formula:

$$\text{ITC}\;(f,t)=\left|\frac{1}{n}\sum_{N=1}^{n}e^{iФ(f,t)}\right|$$ (1)

where *n* is the number of trials, with phase angle *Ф* at trial *N*, at a frequency *f* and time *t*. An ITC value of 0 indicates a uniform distribution of phase angles across trials. An ITC value of 1.0 indicates completely identical phase angles. For displaying the time-frequency power plots shown in Figs 2A, 2B, 3A and 3B, we used EEGLAB (function: newtimef.m) [31]. To extract power spectra, we employed successive overlapping time windows using a three-cycle wavelet at the lowest frequency, continuously expanding to the highest frequency in the range of 3 and 100 Hz (wavelet cycles = [3 0.5]). Baseline power normalization at each frequency was performed during a baseline period of 500 ms prior to the sequence onset. The data were first averaged across trials and decibel-normalized. Significant levels were evaluated by randomly shuffling the spectral estimates from different time windows during the 500-ms baseline period (bootstrap, *p* < 0.05), for details see [31].

#### Phase–amplitude coupling MI

To evaluate the strength of phase–amplitude coupling of the LFPs in the monkey and human data, an MI was calculated between two different frequency ranges of phase and amplitude (Figs 2 and 3). First, the averaged LFP signals (ERP) were subtracted from LFP signals for each trial per sound sequence and this signal was bandpass-filtered using MATLAB’s fir1 and filtfilt functions. For the frequency of amplitude (i.e., gamma), a bandwidth of 20 Hz was used for extracting the analytic amplitude in the frequency range between 40 and 200 Hz in 5-Hz steps. For the frequency of phase, a bandwidth of 1 Hz was used for extracting analytic phase in the frequency range between 3 and 8 Hz in a 1-Hz step. The analytic phase and amplitude were extracted using the Hilbert transform.

To calculate the coupling strength between low-frequency phase and high-frequency amplitude, a composite, complex-valued, time-varying signal *z* was constructed by combining the analytic amplitude of one frequency and the analytic phase of the other using Euler's formula as follows:

$$z(t)=A(t)(e^{iФ(t)}-\bar{Ф})$$ (2)

$$\bar{Ф}=\frac{1}{n}\sum_{t=1}^{n}e^{iФ(t)}$$ (3)

where *n* is the total number of samples, *t* is the time point, *A* is the amplitude of one frequency (e.g., gamma), *Ф* is the phase (in radians) of the other frequency (e.g., theta) after the mean phase angle subtraction (Eq 2). Nonuniformity of phase angle distribution could bias the detection of PAC in the low-frequency band, therefore we performed a linear removal of the phase clustering bias by subtracting the average vector of the phase angles from each phase angle before multiplying them with the power values in the Euler transform [42]. The results were robust to other types of analyses, being qualitatively similar using other approaches such as amplitude-weighted phase locking values. A uniform phase distribution in the complex plane would result from independent amplitude and phase of the time-series. A lack of uniformity results from a dependence between phase and amplitude, which can be measured by the length of the mean vector *z*(*t*) defined as a MI, which is similar to the modulation index used in Canolty et al. [25], as follows:

$${MI}_{raw}=\left|\frac{1}{n}\sum_{t=1}^{n}z(t)\right|$$ (4)

The MI was then normalized by a null distribution of MIs generated from surrogate data lacking the temporal relationships within a trial between phase and power. This null distribution was created by using trial-shuffled composite time series [54] by calculating *Ф*(*t*) from trial *j* and A(*t*) from trial *k*, where *j* and *k* were selected randomly from the total number of trials in each session. Shuffling over trials corrects PAC artefacts arising from concurrent evoked and induced responses that are time-locked to stimulus trial onset, which may not reflect true cross-frequency coupling. Phase locking might come about through evoked responses and high-gamma responses that are both time-locked to the stimulus onset but are otherwise independent of each other, which is a possibility we wanted to exclude. Because shuffling the phase and amplitude components separately over trials does not change the average evoked and induced responses, it should not affect PAC related to these averages; this approach should, however, abolish any contribution of trial-by-trial variability to the PAC. The shuffled data therefore give a null distribution that accounts for potentially spurious phase locking explained by the average responses but otherwise assumes no between-trial PAC. By pairing between phase and amplitude from different trials, this procedure also allows removal of the influence of large-amplitude fluctuations, which might spuriously amplify PAC values. For the null distribution, surrogate MIs were created using 200 permutations, and the raw MIs were transformed to *z* scores by subtracting the mean of the surrogate distribution and then normalizing by its standard deviation. In the absence of phase–amplitude modulation, MI values will vary around 0, whereas MI values significantly greater than 0 reflect phase–amplitude coupling. In addition to shuffling over trials, we used an alternate method to create surrogate data by adding a large temporal offset in one of the time series of the composite signals, as described by Canolty et al. [25]; these two methods generated qualitatively similar results, an observation also noted in the supplementary materials of Tort et al. [54].

We calculated the PAC in response to nonsense words per recording site as follows: the MI matrix was computed during the entire sound sequence duration (2,665 ms after sound onset) for all pairs of amplitude (40–200 Hz in 5-Hz steps) and phase (3–8 Hz in a 1-Hz step). This was done by concatenating across trials to increase the signal-to-noise ratio needed to estimate cross-frequency coupling and detect task-related coupling effects [91]. The statistical significance threshold for MI values was determined using Bonferroni correction for multiple comparisons (α = 0.05, 144 comparisons of pixels in the MI matrix, corresponding to a corrected *p* < 0.05 and *z* score of 3.39 or higher). MI matrices were generated for individual LFP sites with significant PAC.

Time courses of PAC at each pair of frequencies of amplitude and phase from the MI matrix were extracted using the Morlet wavelet analysis. The MI values were calculated using a 30 ms–step time window over the probe stimulus analysis window (1,127 ms) which contains the same acoustic elements for both violation and corresponding consistent sequences to detect transient changes in sequencing context–sensitive neural responses after the violation sound onset or corresponding sounds in the consistent sequence. To increase the signal-to-noise ratio to compensate for using a short time window, we concatenated data from each time window over trials [91]. Then, the same trial shuffle procedure as described above was performed using *z* scores. To evaluate whether the observed PAC is significantly modulated over time by the sequencing context (i.e., violation versus consistent), several criteria were applied: Firstly, the sequencing context effect was evaluated for sites with a significant PAC in response to the nonsense words (Bonferroni correction: 144 pairs of frequencies for phase and amplitude, corresponding to a corrected *p* < 0.05 and *z* = 3.39). This allowed us to ask whether for considerable general PAC effects in the MI matrix (in response to nonsense words across all sequencing conditions) there are differences across the sequencing conditions (consistent versus violation) and whether these are time specific (Bonferroni correction: 38 time points, corresponding to a corrected *p* < 0.05 and *z* = 2.43). Secondly, the mean difference plot of PAC time course values (differential MI values in response to the violation or consistent sequences) needed to deviate from the 95% confidence interval of a null distribution of difference waveforms calculated during the baseline period (200 to 700 ms prior to sound onset). Lastly, sequencing context effects were only evaluated after any preceding differential response, if it was present, to the acoustically different stimuli preceding the probe stimulus window became not significant.

We also calculated the latency of any differential responses to the violation or consistent sequencing context. The sensitivity latency in the PAC response over time was defined as the first time point when the significant sequencing context–dependent response was observed meeting the above-three criteria. Mean latencies in response to violation sequences and consistent sequences were separately calculated per site, provided that more than one sequence pair elicited significant sequencing context–dependent responses at the recording site.

## Supporting information

S1 Fig Artificial grammar sequences and recording sites. A. Artificial grammar exposure and testing sequences. This figure shows the composition of all of the exposure and testing sequences used in these experiments. The letters (A, C, D, F, G) represent the specific nonsense words in the sequences (see manuscript Materials and Methods). First, eight exposure sequences were individually presented in random order. During the subsequent testing phase, one of the eight ‘consistent’ or ‘violation’ sequences was randomly selected for presentation without replacement. Some violation sequences could have multiple violations (bottom-right in panel A), but for this study, analysis was only conducted on effects related to the first violation in the sequences. The monkeys were tested on the two blocks (shown in the right panel in A) separately. Human participants were exposed for 10 mins and tested for 10 mins with all of the exposure or testing sequences, respectively, in one block. Red letters denote the first element after a violation transition in a violation sequence, and the green boxes show the corresponding acoustical elements used for analysis in the comparison consistent sequence pairs. B. Schematic of all pairs of consistent and violation sequences used in the analyses. Shown are the comparison pairs of consistent and violation sequences, highlighting the acoustically matched sections of the sequences used for analysis. Red boxes highlight the element after an illegal violation transition in the violation sequences, also depicted by a red line between elements. All violation sequences are aligned and paired to a matching consistent sequence pair (‘probe stimulus analysis window’ denoted by the black arrays). C. Neuronal response tonotopic maps in Monkey 1 (M1) and Monkey 2 (M2). The color maps depict the best frequency (BF) pure tone responses of the auditory neurons within the recording sites (neurons with tone firing rate responses &gt; 3SD from the baseline no-sound stimulation period; M1: n = 142; M2: n = 160). For display purposes the 1mm grid spacing is smoothed using a 3x3 pixel moving average filter.
(TIF)

S2 Fig Behavioral results on the AGL paradigm in human patient (H1) after the surgical monitoring period. A. Reaction times after offset of the testing sequences for which a correct response was given were significantly shorter in reaction time (RT) to the violation sequences compared to the consistent sequences (consistent: 1.5 ± 0.8 secs in 55 trials out of 80 consistent trials; violation: 1.1 ± 0.6 secs in 46 trials out of 80 violation trials; *p* < 0.001, Mann-Whitney rank test). B. Percent of trials within the 160 trial experiment (80 trials for consistent and violation sequences, respectively) in which the subject responded to the test sequences as ‘different’ to those heard during exposure. We observe a significantly greater response as ‘different’ to the violation sequences (red bar; *p* < 0.01, χ2 = 6.53, χ2 test) than to the consistent sequences (blue bar).
(TIF)

S3 Fig Exemplary ERP, EPR-subtracted LFP, phase-amplitude coupling (PAC), and inter-trial phase coherence (ITC) responses. A. An exemplary averaged monkey LFP response (ERP) to the violation (left column) and the consistent sequence (right column). The horizontal color keys above the response curve identify the time of occurrence of the elements in the sequences. The red vertical lines indicate the onset of the sequence and the blue vertical lines indicate the onset and offset of the probe stimulus period after the violation or corresponding time during the consistent sequence. The bottom panels in A show a magnified view of the ERP response during the probe stimulus analysis window shown. B. An exemplary ERP (blue), raw single-trial LFP (green; nth trial), and ERP-subtracted single-trial LFP (red) response signals during the same probe stimulus window shown in A. C. PAC response to the violation (left) and consistent sequences (right) shown in B. The ERP was subtracted trial-by-trial from the LFP signals prior to PAC analysis (see Materials and Methods). The line plots show the time course of PAC extracted from all the pairs of amplitude and phase of MI matrix in response to the nonsense words, regardless of the sequencing context (*p* < 0.05, Bonferroni correction). The horizontal dotted line denotes the threshold of significance (*p* < 0.05, Bonferroni-corrected). D. (left) PAC response to the violation (pink) and consistent sequences (blue). The examples are the same as shown in C. (right) Difference plot of the time course of PAC response to violation vs. consistent sequences shown in the left panel. Figure format is the same as in manuscript Fig 2D and Fig 3D and 3F. E. Inter-trial phase coherence (ITC) in response to the violation (left) and consistent (right) sequences during the same probe stimulus analysis window as in A-C. If stimulus-driven phase resetting leads to PAC, the ITC shown in E should be the same for the two sequencing conditions, given that the two elements during the probe stimulus analysis window are acoustically identical for both the violation and consistent sequences. Note however that PAC results typically do not show a similar phase clustering in time as the ITC, which is earlier and will pick up stimulus driven phase coherence (compare the results for PAC in panels C to the ITC results in panel E). Thus, the sequencing context elicited PAC appears to be independent of the stimulus-driven time-locked component.
(TIF)

S4 Fig Distributions of frequency of phase and amplitude for the PAC response in humans and monkeys. The distributions of peak PAC values were calculated per phase (A, C) or amplitude (B, D) separately. The error bars denote the standard deviation. No obvious differences are seen between the results in the two monkeys or the two humans.
(TIF)

S5 Fig Topography of PAC frequency of phase and amplitude in human Heschl’s Gyrus. The distributions of peak PAC values were calculated at postero-medial (blue) and antero-lateral (red) recording sites separately per phase (A, C) or amplitude (B, D). The error bars denote the standard deviation. The boundaries in the two subjects between the postero-medial and the antero-lateral aspects of Heschl’s Gyrus are based on the morphology of the short-latency auditory evoked potentials (AEP) to sound click trains and frequency following responses (S1 Text). No obvious topographical differences between PAC responses in postero-medial versus antero-lateral HG are seen.
(TIFF)

S6 Fig Tonotopic maps and locations of significant sequencing context effects. A. Tonotopic maps based on fMRI (left) and electrophysiological recordings (right) of two animals. The fMRI image on the left shows a slice looking down on the supratemporal plane. B. Recording sites that showed sequencing context effects across all LFP signals (theta, low-gamma, and high-gamma) and SUA. The color denotes the number of sequences that elicited significant contextual responses. The results do not show a particular clustering in the amount of significant responses sensitive to the sequencing context.
(TIF)

S7 Fig Control experiment in human participant H3 and resulting PAC effects. A. Time course of the experiments. The testing conditions were identical, with the key difference what the subject experienced before testing: either exposure to random transitions between the nonsense words in a sequence or structured sequences consistent with the artificial grammar learning (AGL) paradigm, see manuscript text and S1 Text for further details. B. Reconstructed image of the location of the depth electrode placement on the left HG for H3. C-D. Resulting PAC response during the first testing session after exposure to random transitions in the sequences. The majority of sites (3/5) showed significant phase-amplitude coupling (PAC) in response to the nonsense words. An exemplary response is shown in C for channel 57, whereas the majority of the sites (4/5) showed no significant sequencing context effect (consistent vs. violation, see exemplary PAC response in D). Figure format is the same as in Fig 2C and 2D and Fig 3C–3F. Resulting PAC responses during the second testing session after exposure to legal AGL sequences (same as the ones used in the main experiment reported in the manuscript: see exposure AGL set of sequences in the Materials and Methods). All of the sites (5/5) showed significant PAC in response to the nonsense words (an exemplary response is shown in E for channel 60). The majority of the sites (3/5) also showed significant sequencing context effects (an exemplary response is shown in F for channel 56 where a significant consistent sequence preference is seen with a sensitivity latency of 900 ms (the earlier violation sequence sensitivity did not breach for long enough to be significant by the joint magnitude and duration criteria (see Materials and Methods).
(TIF)

S8 Fig Correlation analysis between the magnitude of the response to the sounds preceding the probe stimulus window (x-axis) and the magnitude of the sequencing context effect during the probe stimulus (y-axis) for LFP power. The data from high-gamma, low-gamma, and theta bands are displayed here together as the results were comparable for the separate frequency bands. No significant correlations were seen in these analyses (all *p* > 0.1) between the sequencing context response and the magnitude of the response to the acoustically different sounds preceding the probe stimulus (where the acoustical items were identical and during which the sequencing context response was calculated).
(TIFF)

S9 Fig Monkey and human auditory cortex fMRI responses (data re-analyzed from Wilson et al., Nature Communications, 2015 [20]) do not show strong sequencing context sensitivity. The analyses performed in Wilson et al. (2015 [20]) report no significant activation to the violation vs consistent contrast within auditory cortex in either the macaque or human data at a cluster corrected significance threshold (*p* < 0.05). Here we looked for subthreshold sensitivity, as follows: A. Mean activation (Z-score) to the violation vs consistent contrast was calculated across primary auditory cortex (field A1) for each of the macaques. These analyses revealed limited and variable activation patterns across the macaques tested, and none of the macaques showed differential activation to the violation vs consistent sequences that exceeded even a very liberal significance threshold (uncorrected *p* = 0.05 corresponding to Z = 1.96; see dotted lines). M1 is the same animal studied in this electrophysiological report. B. The location of the anatomical ROI used for these analyses in the macaque auditory cortex (field A1). C. Human medial Heschl’s gyrus also showed no significant differential activation to the violation vs consistent contrast. D. Location of the anatomical ROI used in the human fMRI data analyses. See manuscript text for discussion.
(TIF)

S1 Table Control experiment in human participant H3 and resulting PAC effects. Number of sites with significant PAC responses and sequencing context PAC effects during the same type of testing after exposure to sequences containing random transitions between the nonsense words.
(XLSX)

S2 Table Control experiment in human participant H3 and resulting PAC effects. Number of sites with significant PAC responses and sequencing context PAC effects during the same type of testing phase after exposure to structured sequences consistent with the AG ordering relationships.
(XLSX)

S1 Text Lack of relationship between sequencing context sensitive neural effects and auditory cortex topography. (DOCX)

S2 Text Exposure to random transitions or structured sequence ordering relationships: Control experiment in human participant (H3). (DOCX)

S3 Text Effects of the acoustical elements preceding the probe stimulus analysis window. (DOCX)

## Abbreviations

EEG: electroencephalography
ERSP: event-related spectral perturbation
fMRI: functional magnetic resonance imaging
HG: Heschl’s gyrus
ISI: interstimulus interval
ITC: intertrial phase coherence
ITI: intertrial interval
LFP: local field potential
MEG: magnetoencephalography
MI: modulation index
PAC: phase–amplitude coupling
SSA: stimulus-specific adaptation
SUA: single-unit activity
