# Supplementary figures and images for: Sequence learning modulates neural responses and oscillatory coupling in human and monkey auditory cortex
Source: PLoS Biol. 2017 Apr 25;15(4):e2000219. doi: 10.1371/journal.pbio.2000219 (PMC5404755; doi:10.1371/journal.pbio.2000219)

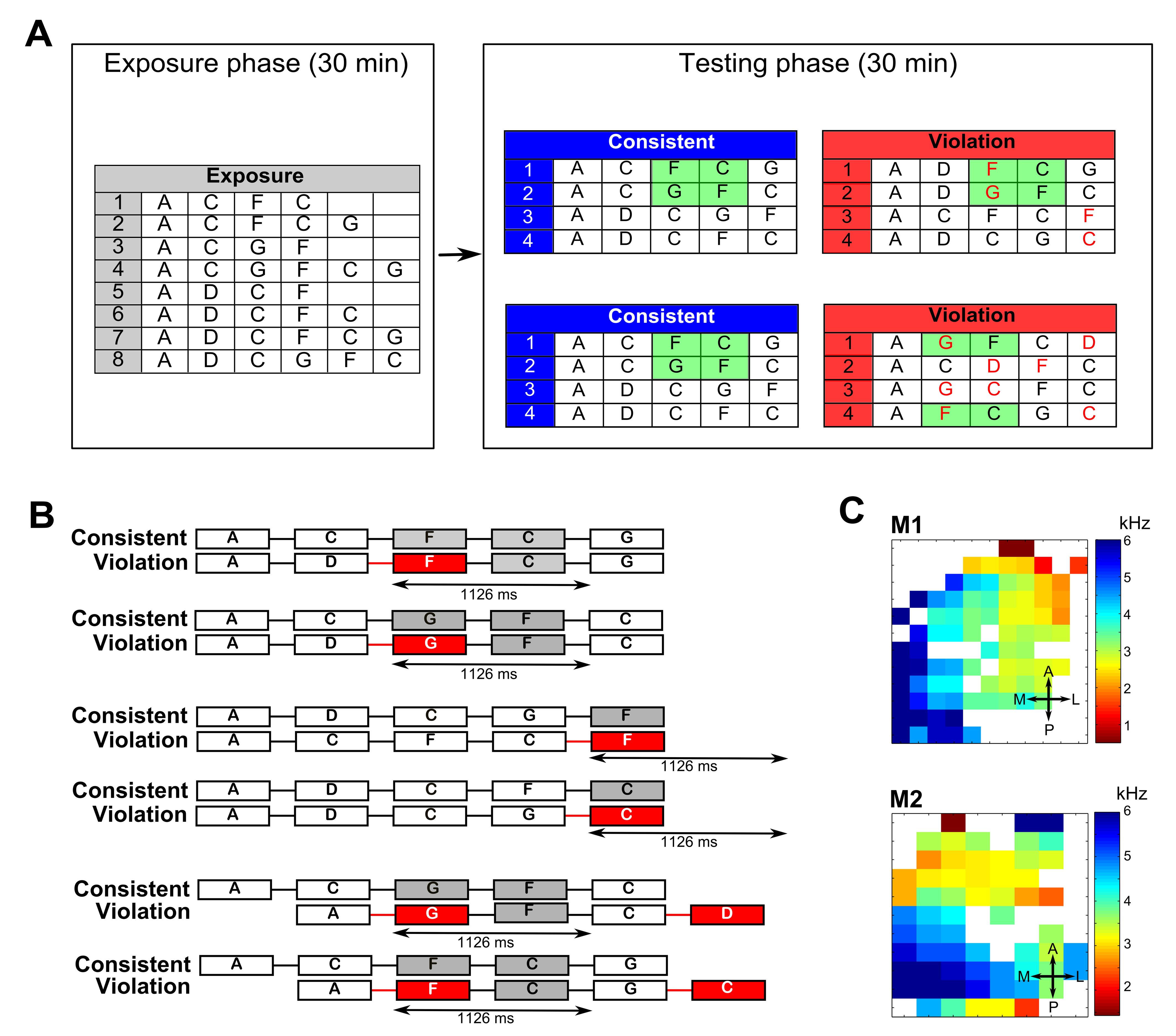

Supplement: S1 Fig — A. Artificial grammar exposure and testing sequences. This figure shows the composition of all of the exposure and testing sequences used in these experiments. The letters (A, C, D, F, G) represent the specific nonsense words in the sequences (see manuscript Materials and Methods). First, eight exposure sequences were individually presented in random order. During the subsequent testing phase, one of the eight ‘consistent’ or ‘violation’ sequences was randomly selected for presentation without replacement. Some violation sequences could have multiple violations (bottom-right in panel A), but for this study, analysis was only conducted on effects related to the first violation in the sequences. The monkeys were tested on the two blocks (shown in the right panel in A) separately. Human participants were exposed for 10 mins and tested for 10 mins with all of the exposure or testing sequences, respectively, in one block. Red letters denote the first element after a violation transition in a violation sequence, and the green boxes show the corresponding acoustical elements used for analysis in the comparison consistent sequence pairs. B. Schematic of all pairs of consistent and violation sequences used in the analyses. Shown are the comparison pairs of consistent and violation sequences, highlighting the acoustically matched sections of the sequences used for analysis. Red boxes highlight the element after an illegal violation transition in the violation sequences, also depicted by a red line between elements. All violation sequences are aligned and paired to a matching consistent sequence pair (‘probe stimulus analysis window’ denoted by the black arrays). C. Neuronal response tonotopic maps in Monkey 1 (M1) and Monkey 2 (M2). The color maps depict the best frequency (BF) pure tone responses of the auditory neurons within the recording sites (neurons with tone firing rate responses > 3SD from the baseline no-sound stimulation period; M1: n = 142; M2: n = 160). For di [file pbio.2000219.s001.tif]

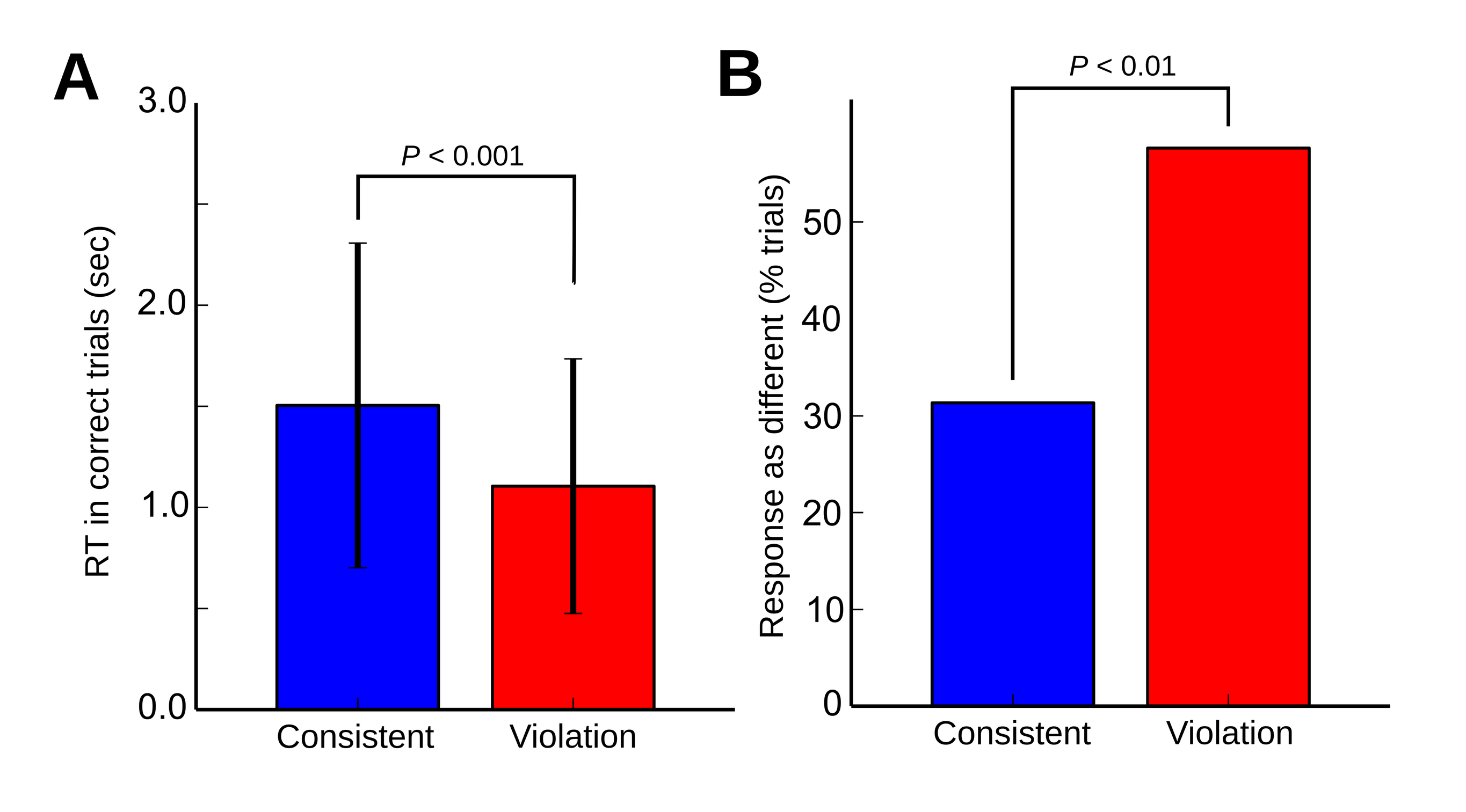

Supplement: S2 Fig — A. Reaction times after offset of the testing sequences for which a correct response was given were significantly shorter in reaction time (RT) to the violation sequences compared to the consistent sequences (consistent: 1.5 ± 0.8 secs in 55 trials out of 80 consistent trials; violation: 1.1 ± 0.6 secs in 46 trials out of 80 violation trials; p < 0.001, Mann-Whitney rank test). B. Percent of trials within the 160 trial experiment (80 trials for consistent and violation sequences, respectively) in which the subject responded to the test sequences as ‘different’ to those heard during exposure. We observe a significantly greater response as ‘different’ to the violation sequences (red bar; p < 0.01, χ2 = 6.53, χ2 test) than to the consistent sequences (blue bar). (TIF) [file pbio.2000219.s002.tif]

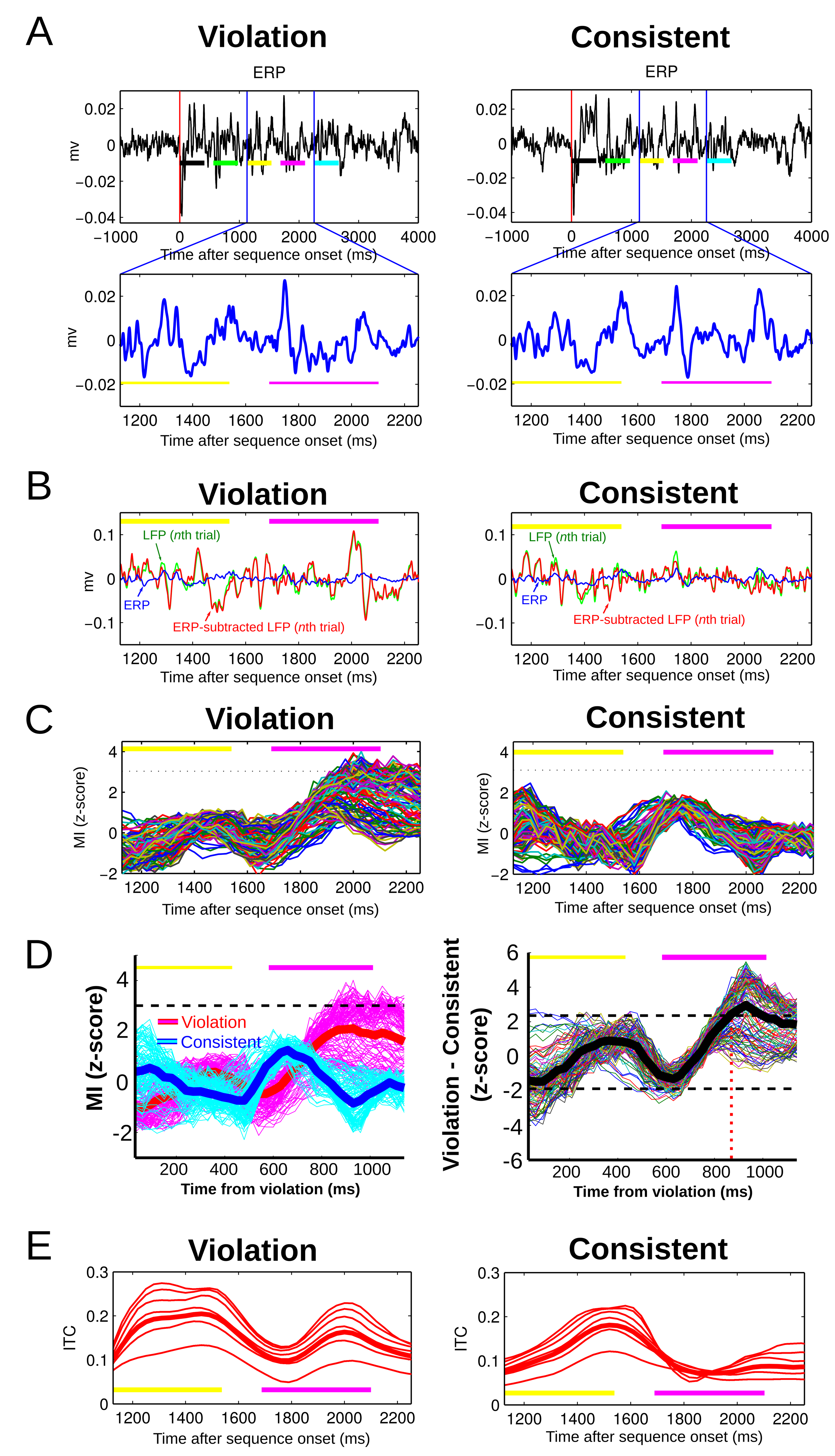

Supplement: S3 Fig — A. An exemplary averaged monkey LFP response (ERP) to the violation (left column) and the consistent sequence (right column). The horizontal color keys above the response curve identify the time of occurrence of the elements in the sequences. The red vertical lines indicate the onset of the sequence and the blue vertical lines indicate the onset and offset of the probe stimulus period after the violation or corresponding time during the consistent sequence. The bottom panels in A show a magnified view of the ERP response during the probe stimulus analysis window shown. B. An exemplary ERP (blue), raw single-trial LFP (green; nth trial), and ERP-subtracted single-trial LFP (red) response signals during the same probe stimulus window shown in A. C. PAC response to the violation (left) and consistent sequences (right) shown in B. The ERP was subtracted trial-by-trial from the LFP signals prior to PAC analysis (see Materials and Methods). The line plots show the time course of PAC extracted from all the pairs of amplitude and phase of MI matrix in response to the nonsense words, regardless of the sequencing context (p < 0.05, Bonferroni correction). The horizontal dotted line denotes the threshold of significance (p < 0.05, Bonferroni-corrected). D. (left) PAC response to the violation (pink) and consistent sequences (blue). The examples are the same as shown in C. (right) Difference plot of the time course of PAC response to violation vs. consistent sequences shown in the left panel. Figure format is the same as in manuscript Fig 2D and Fig 3D and 3F. E. Inter-trial phase coherence (ITC) in response to the violation (left) and consistent (right) sequences during the same probe stimulus analysis window as in A-C. If stimulus-driven phase resetting leads to PAC, the ITC shown in E should be the same for the two sequencing conditions, given that the two elements during the probe stimulus analysis window are acoustically identical for both the violation and consistent sequ [file pbio.2000219.s003.tif]

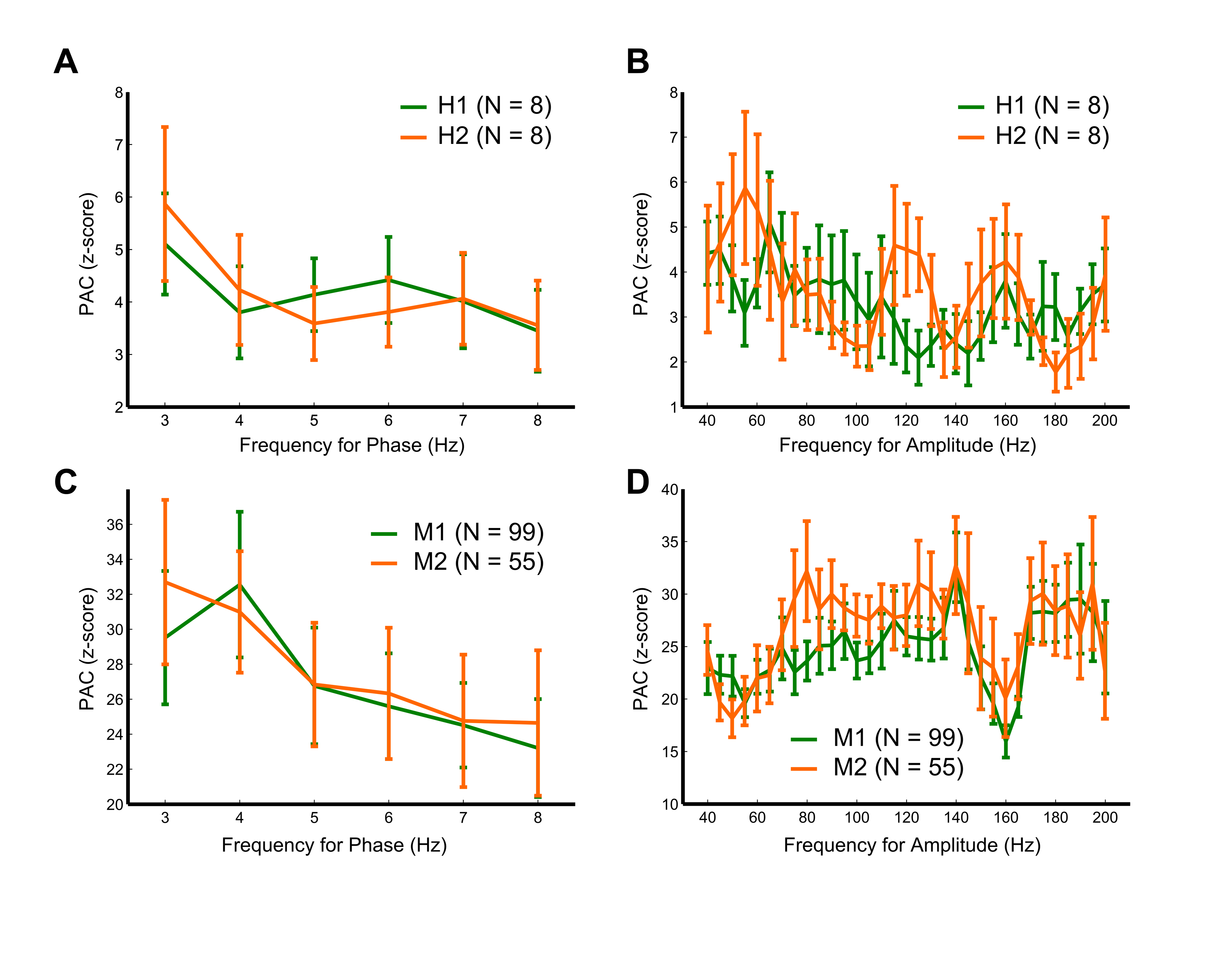

Supplement: S4 Fig — The distributions of peak PAC values were calculated per phase (A, C) or amplitude (B, D) separately. The error bars denote the standard deviation. No obvious differences are seen between the results in the two monkeys or the two humans. (TIF) [file pbio.2000219.s004.tif]

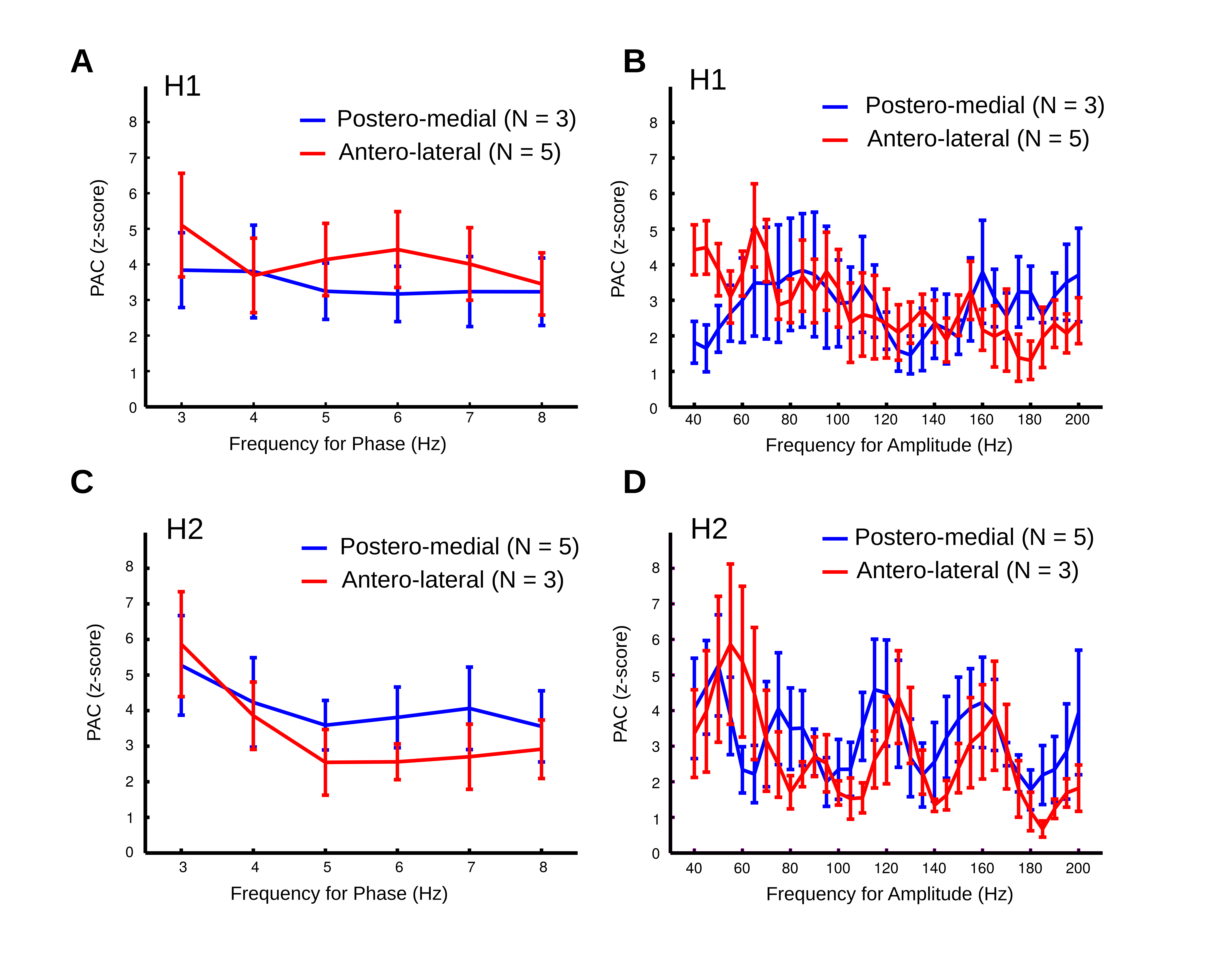

Supplement: S5 Fig — The distributions of peak PAC values were calculated at postero-medial (blue) and antero-lateral (red) recording sites separately per phase (A, C) or amplitude (B, D). The error bars denote the standard deviation. The boundaries in the two subjects between the postero-medial and the antero-lateral aspects of Heschl’s Gyrus are based on the morphology of the short-latency auditory evoked potentials (AEP) to sound click trains and frequency following responses (S1 Text). No obvious topographical differences between PAC responses in postero-medial versus antero-lateral HG are seen. (TIFF) [file pbio.2000219.s005.tiff]

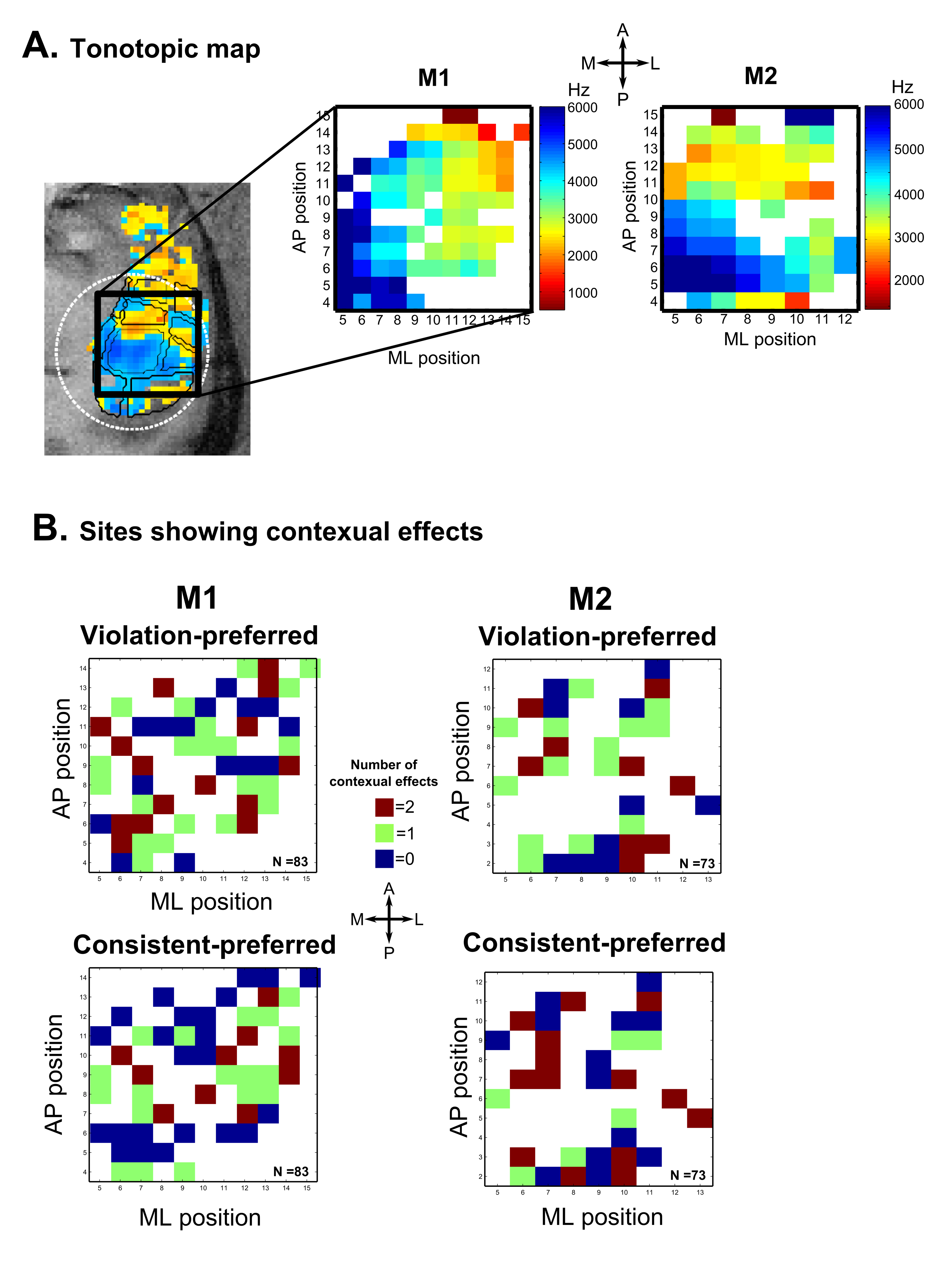

Supplement: S6 Fig — A. Tonotopic maps based on fMRI (left) and electrophysiological recordings (right) of two animals. The fMRI image on the left shows a slice looking down on the supratemporal plane. B. Recording sites that showed sequencing context effects across all LFP signals (theta, low-gamma, and high-gamma) and SUA. The color denotes the number of sequences that elicited significant contextual responses. The results do not show a particular clustering in the amount of significant responses sensitive to the sequencing context. (TIF) [file pbio.2000219.s006.tif]

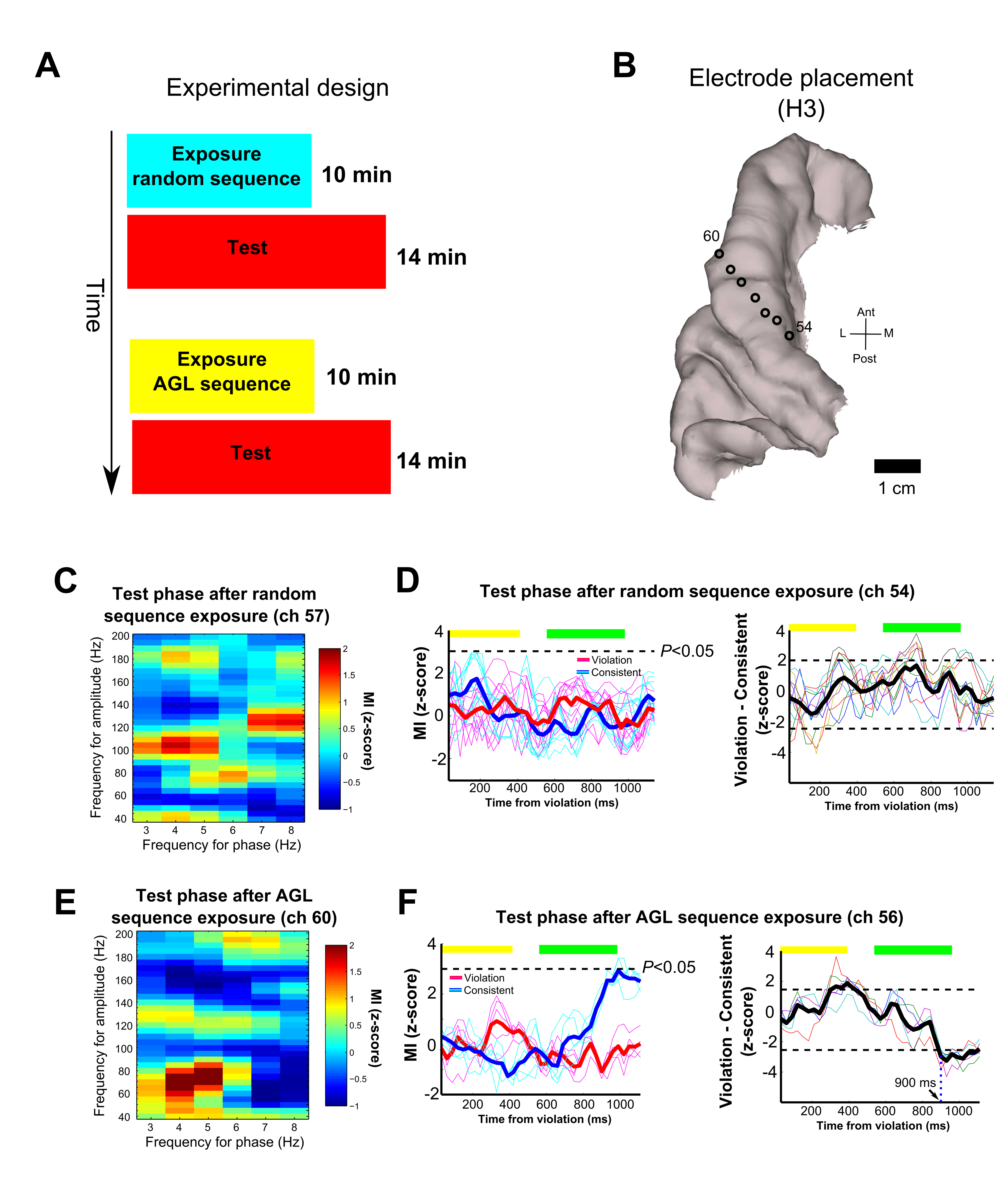

Supplement: S7 Fig — A. Time course of the experiments. The testing conditions were identical, with the key difference what the subject experienced before testing: either exposure to random transitions between the nonsense words in a sequence or structured sequences consistent with the artificial grammar learning (AGL) paradigm, see manuscript text and S1 Text for further details. B. Reconstructed image of the location of the depth electrode placement on the left HG for H3. C-D. Resulting PAC response during the first testing session after exposure to random transitions in the sequences. The majority of sites (3/5) showed significant phase-amplitude coupling (PAC) in response to the nonsense words. An exemplary response is shown in C for channel 57, whereas the majority of the sites (4/5) showed no significant sequencing context effect (consistent vs. violation, see exemplary PAC response in D). Figure format is the same as in Fig 2C and 2D and Fig 3C–3F. Resulting PAC responses during the second testing session after exposure to legal AGL sequences (same as the ones used in the main experiment reported in the manuscript: see exposure AGL set of sequences in the Materials and Methods). All of the sites (5/5) showed significant PAC in response to the nonsense words (an exemplary response is shown in E for channel 60). The majority of the sites (3/5) also showed significant sequencing context effects (an exemplary response is shown in F for channel 56 where a significant consistent sequence preference is seen with a sensitivity latency of 900 ms (the earlier violation sequence sensitivity did not breach for long enough to be significant by the joint magnitude and duration criteria (see Materials and Methods). (TIF) [file pbio.2000219.s007.tif]

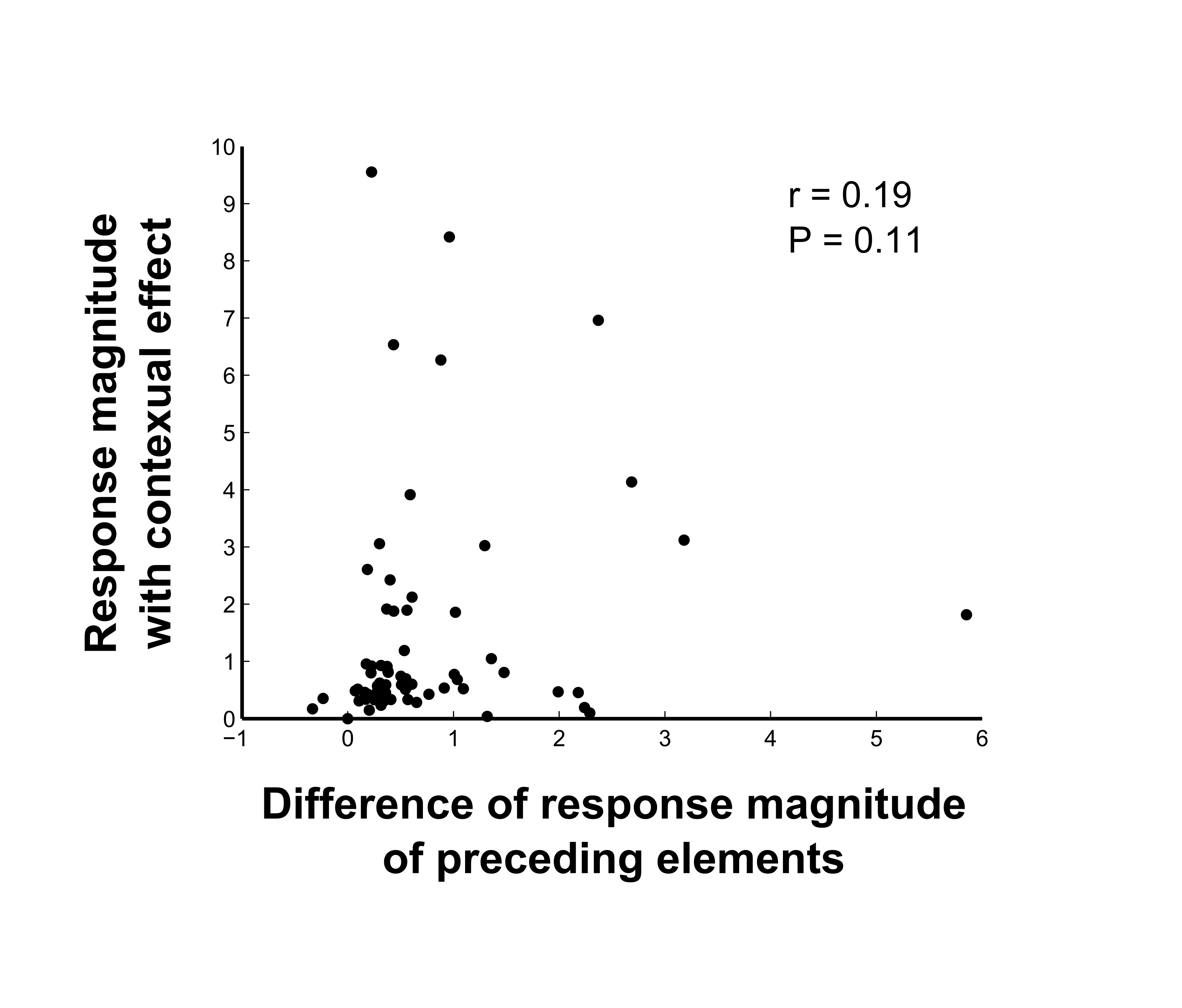

Supplement: S8 Fig — The data from high-gamma, low-gamma, and theta bands are displayed here together as the results were comparable for the separate frequency bands. No significant correlations were seen in these analyses (all p > 0.1) between the sequencing context response and the magnitude of the response to the acoustically different sounds preceding the probe stimulus (where the acoustical items were identical and during which the sequencing context response was calculated). (TIFF) [file pbio.2000219.s008.tiff]

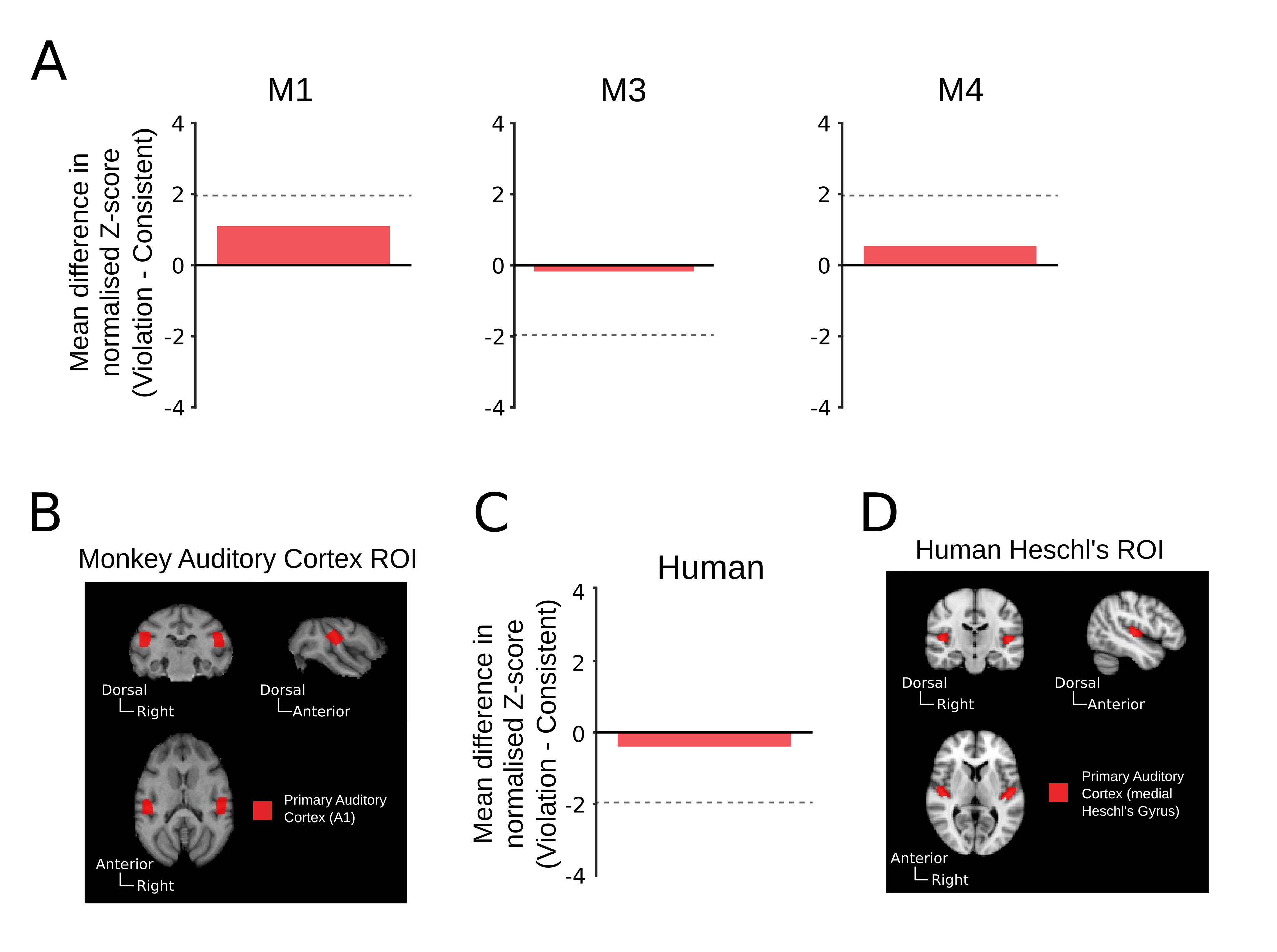

Supplement: S9 Fig — The analyses performed in Wilson et al. (2015 [20]) report no significant activation to the violation vs consistent contrast within auditory cortex in either the macaque or human data at a cluster corrected significance threshold (p < 0.05). Here we looked for subthreshold sensitivity, as follows: A. Mean activation (Z-score) to the violation vs consistent contrast was calculated across primary auditory cortex (field A1) for each of the macaques. These analyses revealed limited and variable activation patterns across the macaques tested, and none of the macaques showed differential activation to the violation vs consistent sequences that exceeded even a very liberal significance threshold (uncorrected p = 0.05 corresponding to Z = 1.96; see dotted lines). M1 is the same animal studied in this electrophysiological report. B. The location of the anatomical ROI used for these analyses in the macaque auditory cortex (field A1). C. Human medial Heschl’s gyrus also showed no significant differential activation to the violation vs consistent contrast. D. Location of the anatomical ROI used in the human fMRI data analyses. See manuscript text for discussion. (TIF) [file pbio.2000219.s009.tif]
