# Supplementary material for: Sequence learning modulates neural responses and oscillatory coupling in human and monkey auditory cortex
Source: PLoS Biol. 2017 Apr 25;15(4):e2000219. doi: 10.1371/journal.pbio.2000219 (PMC5404755; doi:10.1371/journal.pbio.2000219)
Supplement: S1 Text — (DOCX) [file pbio.2000219.s012.docx]

# Lack of relationship between sequencing context sensitive neural effects and auditory cortex topography

## Distribution of PAC phase and amplitude effects in humans and monkeys

We first evaluated the distribution of frequency for phase and amplitude in the reported PAC effects. As shown in S4 Fig, there is no clear difference in distribution of the frequency range where the peak PAC effects are observed in the humans or monkeys, showing a low-frequency maximum around 3-4 Hz for phase (A and C). The effects were evident throughout the low- and high-gamma range in LFP amplitude (B and D). Trivially, the coupling strength is generally greater in the monkey data than in humans, because of the greater number of stimulus trials available for analysis in the monkey experiments compared to the data from the patients (see manuscript Materials and Methods).

## Topography of contextual effects between violation and consistent sequences in human Heschl’s gyrus

To examine whether there is any obvious systematic relationship with anatomical topography for the PAC effects in the gamma-band frequencies (i.e., high-gamma or low-gamma), the peak PACs at the medial half of sites (H1: N=3, H2: N=5) and the lateral half of sites (H1: N=5, H2: N=3) along the human HG was evaluated in relation to the anatomical location (S5 Fig, [[1](#_ENREF_1)]). The results do not show a consistent topographical relationship for gamma frequency.

## Tonotopic maps and locations of significant sequencing context effects

We also evaluated the anatomical distribution of contextual effects between violation-preferring and consistent-preferring responses in relation to the tonotopic fMRI and neuronal response maps. S6 Fig. shows the number of sequences that elicited sequencing context sensitive responses across all neural response data (LFP theta, low-gamma, high-gamma) and SUA. Within the monkey auditory cortex sites that we recorded from, no clear topographical relationship is observed that is disproportionately differently sensitive to the violation- and consistent-sequences.

Reference

1. Nourski KV, Steinschneider M, Rhone AE. Electrocorticographic Activation within Human Auditory Cortex during Dialog-Based Language and Cognitive Testing. Frontiers in Human Neuroscience. 2016;10. doi: 10.3389/fnhum.2016.00202.
