# Supplementary material for: Sequence learning modulates neural responses and oscillatory coupling in human and monkey auditory cortex
Source: PLoS Biol. 2017 Apr 25;15(4):e2000219. doi: 10.1371/journal.pbio.2000219 (PMC5404755; doi:10.1371/journal.pbio.2000219)
Supplement: S2 Text — (DOCX) [file pbio.2000219.s013.docx]

# Exposure to random transitions or structured sequence ordering relationships: Control experiment in human participant (H3)

For this control experiment, we generated 125 exposure sequences with a flat transitional probability distribution between the five nonsense words. Each sequence was five element long and each element occurred with exactly the same frequency across the exposure sequences. In these sequences, the transition between two elements was set to be approximately equal across all possible pairs, and, as in the main experiment, the same element was never repeated consecutively in a sequence. Unlike the exposure sequences used in the main experiment, these sequences provide no ordering cues for the subject to learn the AG sequence ordering relationships. The control experiment is a similar to one we used as a control in a monkey in our previous fMRI study [[1](#_ENREF_1)].

The experiment consisted of a 10 minute exposure session using randomly shuffled sequences followed by a 14 minute testing session. During the testing session the same testing sequences that constitute consistent and violation sequences were used as in the main experiments reported in the paper. After a brief break, the subject then was exposed and tested identically as in the main experiment, involving a 10 minutes exposure session with the regular set of exposure sequences generated by the AG, followed by the 14 minute testing session (S7 Fig).

We predict that if the PAC contextual effects reported in this study were affected by structured sequence learning, then the effects should be weaker as a result of exposure with sequences containing random transitions between the elements. It was not possible to counter balance with structured learning occurring first, since it is not known how long the learning effects would carry over. So we opted to begin the experiment with the randomized exposure sequences followed by testing and then the exposure and testing with the structured sequences. However, a general effect of the amount of exposure with any type of sequence order (random or structured) is unlikely to have the specific effects on sequence-context sensitivity in the PAC that were observed.

**Results.** S1 Table and S2 Table show that out of 7 sites in Heschl’s gyrus of the subject (H3), two sites were identified as dead channels (shown as N/A in S1 Table and S2 Table). We found that a majority of sites (3 out of 5) show a significant PAC response to the nonsense words in the testing session after the subject was exposed to the randomly shuffled sequences, but only a minority of sites (1 out of 5) show a sequencing context PAC effect with the random sequences exposure. By contrast, with the structured sequences exposure, all sites (5/5) showed a significant PAC response to the nonsense words and a majority (3/5) were sensitive to the sequence ordering context in the testing sequences. Note that the testing sequences were identical as were the analyses in response to these, with the key difference being what the participant was exposed to prior to testing (random or structured sequences).

Reference

1. Wilson B, Kikuchi Y, Sun L, Hunter D, Dick F, Smith K, et al. Auditory sequence processing reveals evolutionarily conserved regions of frontal cortex in macaques and humans. Nature Communications. 2015;6:8901. doi: 10.1038/ncomms9901.
