# Supplementary material for: Sequence learning modulates neural responses and oscillatory coupling in human and monkey auditory cortex
Source: PLoS Biol. 2017 Apr 25;15(4):e2000219. doi: 10.1371/journal.pbio.2000219 (PMC5404755; doi:10.1371/journal.pbio.2000219)
Supplement: S3 Text — (DOCX) [file pbio.2000219.s014.docx]

# Effects of the acoustical elements preceding the probe stimulus analysis window

To evaluate whether the observed sequencing context-sensitive responses depend on the differential response to the two sounds prior to the violation (after which occurs the probe stimulus period used for the analyses reported in the manuscript), we performed an independent analysis of the LFP and the SUA in response to the single-violation sequences. In this analysis, the difference plot was calculated (violation-consistent) and the peak magnitude extracted at the time point where a maximum response was elicited during the pre-violation period (1 element + 1 ISI = 563ms analysis window). This response was compared to the peak magnitude during the probe stimulus analysis window, after a time point when a sensitive latency was measured as described in the manuscript.

The results for the LFP signal are summarized and illustrated in the S8 Fig. The correlations between responses to the preceding elements prior to the violation and the peak of the significant contextual effect during the probe stimulus window do not show significant associations, apart from low gamma (Pearson correlation: all LFP measures combined: *r* = 0.19, *p* = 0.11; theta: *r* = 0.10, *p* = 0.65; low-gamma, *r* = 0.57, *p* = 0.002; high-gamma: *r* = 0.14, *p* = 0.54). These findings suggest that the reported contextual effects are generally not driven by the LFP response to the acoustically different sounds prior to the violation.

There was a significant association for SUA responses (*r* = 0.50, *p* = 0.0002), which may indicate that the sequence-sensitive effects in single neurons are associated with acoustically driven effects prior to the violation. Yet, this interpretation should be made with caution given that neural effects associated with sound acoustics to the preceding elements are not evident in the LFP responses apart from low gamma which is also difficult to interpret given the general lack of effect in the other LFP frequency bands including high gamma (*p* > 0.5). Moreover, in the EEG signal in our prior study using a similar analysis there was no association between the EEG signal in response to the acoustical elements prior to the violation transition and the reported sequencing-context sensitive effects during the probe stimulus period [[1](#_ENREF_1)]. Finally, for both LFP and SUA, the reported effects in this manuscript were only accepted after the differential responses to the preceding sounds dissipated which should minimize effects in response to the prior acoustical elements (see Materials and Methods for details). In summary, effects of responses to the acoustical elements preceding the probe stimulus analysis window are variable at best.

Reference

1. Attaheri A, Kikuchi Y, Milne AE, Wilson B, Alter K, Petkov CI. EEG potentials associated with artificial grammar learning in the primate brain. Brain and Language. 2014. doi: 10.1016/j.bandl.2014.11.006.
